# Identification of Polo-like kinase 1 interaction inhibitors using a novel cell-based assay

**DOI:** 10.1038/srep37581

**Published:** 2016-11-22

**Authors:** Karine Normandin, Jean-François Lavallée, Marie Futter, Alexandre Beautrait, Jean Duchaine, Sébastien Guiral, Anne Marinier, Vincent Archambault

**Affiliations:** 1 Institute for Research in Immunology and Cancer, Université de Montréal, Montréal, Canada; 2 Département de chimie, Université de Montréal, Montréal, Canada; 3 Département de biochimie et médecine moléculaire, Université de Montréal, Montréal, Canada.

## Abstract

Polo-like kinase 1 (Plk1) plays several roles in cell division and it is a recognized cancer drug target. Plk1 levels are elevated in cancer and several types of cancer cells are hypersensitive to Plk1 inhibition. Small molecule inhibitors of the kinase domain (KD) of Plk1 have been developed. Their selectivity is limited, which likely contributes to their toxicity. Polo-like kinases are characterized by a Polo-Box Domain (PBD), which mediates interactions with phosphorylation substrates or regulators. Inhibition of the PBD could allow better selectivity or result in different effects than inhibition of the KD. *In vitro* screens have been used to identify PBD inhibitors with mixed results. We developed the first cell-based assay to screen for PBD inhibitors, using Bioluminescence Resonance Energy Transfer (BRET). We screened through 112 983 compounds and characterized hits in secondary biochemical and biological assays. Subsequent Structure-Activity Relationship (SAR) analysis on our most promising hit revealed that it requires an alkylating function for its activity. In addition, we show that the previously reported PBD inhibitors thymoquinone and Poloxin are also alkylating agents. Our cell-based assay is a promising tool for the identification of new PBD inhibitors with more drug-like profiles using larger and more diverse chemical libraries.

Polo-like kinase 1 (Plk1) is an essential cell cycle regulator and a target for cancer drug development. Discovered in Drosophila as a gene required for mitosis, the Polo kinase was found to have orthologs in most eukaryotes ranging from yeasts to humans^1^,^2^,^3^. In humans, there exist 5 members of the Polo-like kinase (Plk) family^4^. Plks are serine/threonine kinases characterized by the presence of a C-terminal Polo-Box Domain (PBD), which mediates protein interactions with substrates and regulators^5^,^6^. Plk1 plays multiple essential as well as non-essential roles in the regulation of cell division. Plk1 is required for several aspects of centrosome function, chromosome dynamics in mitosis, spindle function and cytokinesis^3^,^6^,^7^.

Hundreds of studies now link Plks to cancer in many different ways. Plk1 is overexpressed in a very broad range of cancers, and a high expression of Plk1 correlates with bad prognosis^8^,^9^,^10^. Many cancer cell types are more sensitive than healthy cells to a reduction in Plk1 function, and this difference defines a theoretically exploitable therapeutic window. Ras-transformed and p53-deficient cells were found to be particularly addicted to high Plk1 activity^11^,^12^,^13^,^14^. For these reasons, Plk1 is recognized as a prime target for anti-cancer drug development. While Plk1 promotes proliferation, Plk2 and Plk3 are involved in cell cycle checkpoints that have anti-proliferative activity and have been proposed to function as tumor suppressors^15^,^16^,^17^,^18^.

Inhibitors of the Plk1 KD have been developed, and some of them are in clinical trials^19^,^20^. However, because the KD is highly conserved among kinases, it has been difficult to achieve selectivity for Plk1, especially with respect to other Plk family members^21^. BI2536 and its derivative volasertib (BI6727) are currently seen as the most promising Plk1 inhibitors^22^,^23^. Volasertib is in phase III clinical trials and has given promising results, most notably for the treatment of acute myeloid leukemia (AML), a devastating disease for which new drugs are desperately needed^24^. In 2013, volasertib received the designation “Breakthrough Therapy” by the FDA. These developments provide a strong validation for Plk1 as a cancer drug target. However, toxicity remains problematic^24^. BI2536 and volasertib inhibit Plk1, Plk2 and Plk3 with similar IC_50_s, as well as other kinases^22^,^23^,^25^. However, because Plk2 and Plk3 have functions akin to tumor suppression, it is desirable to inhibit Plk1 and not other Plks.

Inhibition of the PBD is an alternative and attractive strategy to interfere with Plk1 function. A functional PBD in Plk1 is essential for cell division^26^,^27^,^28^. It allows Plk1 to bind several of its substrates and to be recruited to different locations where its activity is required, including centrosomes, kinetochores and the central spindle in cytokinesis^3^,^29^,^30^. The PBD is unique to Plk members (unlike the KD) and it differs more between Plks in terms of sequence and structure than the KD does. Moreover, Plks have been shown to differ in their preferred phosphopeptide binding motifs^6^,^31^,^32^. Thus, the PBD may be more amenable to the development of Plk1-specific inhibitors than the KD. Besides, some functions of Plk1 may be more reliant on the KD than the PBD, while other functions may depend more on the PBD than the KD. Therefore, inhibition of the PBD may result in different functional consequences than inhibition of the KD and this difference could be advantageous in cancer treatments^33^. Finally, inhibitors of the PBD could act additively or synergistically with KD inhibitors, possibly resulting in an increased efficacy for either compound at tolerable doses.

For these reasons, efforts have already been deployed to develop PBD inhibitors of Plk1. The first proof of principle of this strategy came with the identification of Poloxin and its natural analog thymoquinone (TQ) as PBD inhibitors in an *in vitro* screen using a Fluorescence Polarization assay monitoring the interaction between recombinant PBD and a fluorophore-coupled phosphopeptide^34^,^35^. These compounds inhibited the PBD at low micromolar concentrations *in vitro*. However, high micromolar concentrations were required to disrupt mitosis and induce apoptosis. PBD crystals soaked with TQ revealed an X-ray structure with TQ non-covalently bound to the phosphopeptide binding site of the PBD^36^. Another screen, using an *in vitro* assay monitoring the binding of the PBD tagged with a fluorescent protein to an immobilized phosphopeptide, allowed the identification of purpurogallin (PPG) as a PBD inhibitor^37^. This compound too was active at low micromolar concentrations *in vitro* but impacted mitosis at high micromolar concentrations. The most potent chemical inhibitors of the PBD are phosphopeptides or peptide-like molecules. Burke, Lee and co-workers have made impressive progress to increase the potency and specificity of these compounds^38^,^39^. However, cell permeability of these molecules will remain problematic unless an engineered approach succeeds in efficiently delivering them inside cells^40^,^41^.

We reasoned that a cell-based assay would be advantageous for the identification of PBD inhibitors. It would allow the direct detection of cell-permeable compounds active in an intracellular environment as primary hits in a chemical screen. We have thus developed the first cell-based assay for the identification of PBD interaction inhibitors. Using this assay, we have conducted a HTS screen and identified compounds that we characterized in secondary assays and chemical analyses. We found that our most potent hit acts by alkylation on nucleophilic amino acid side chains, suggesting little potential for drug development. In addition, we show that TQ and Poloxin are also alkylators.

## Results

### A novel cell-based assay to search for PBD interaction inhibitors of Plk1

**Figure 1:**
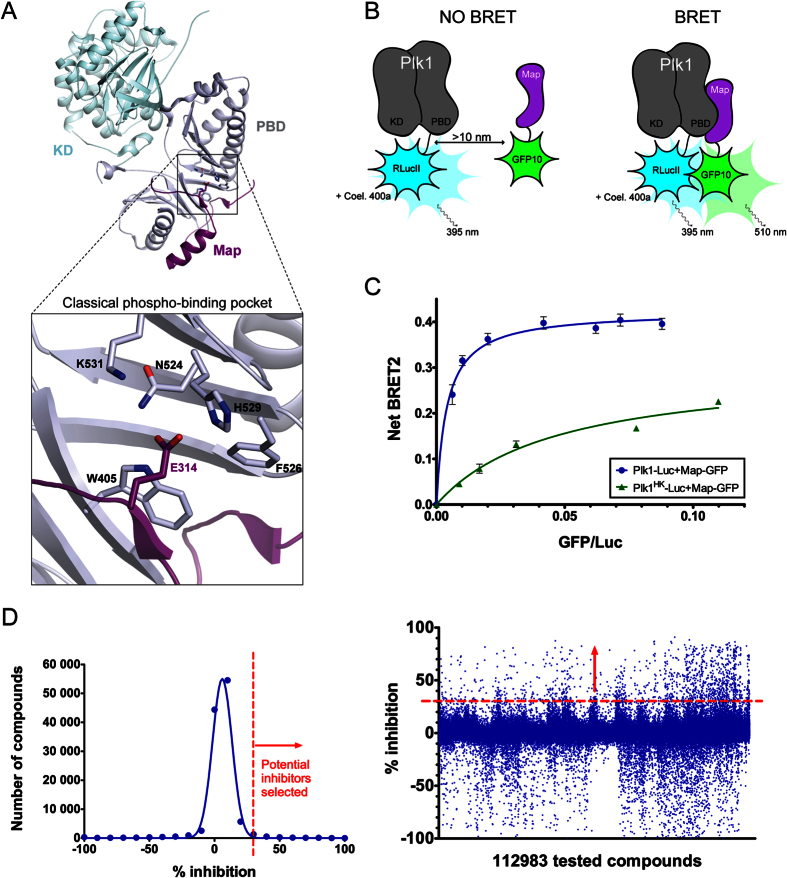
A BRET-based assay to screen for chemical inhibitors of Plk1 protein interaction. (**A**) Co-crystal structure of zebrafish Plk1 (kinase domain and Polo-Box Domain in light blue and grey, respectively) in complex with a fragment of Drosophila Map205 (purple) (PDB entry 4J7B). The assay developed in this study exploited the analogous interaction between human Plk1 and a Drosophila Map205 fragment. Residues of the PBD phospho-binding pocket (zoomed in) within a 4-angstroms distance of Map205 phospho-mimetic residue E314 are shown as sticks (W405 corresponds to W414 in human Plk1, N514 to N523, F526 to F535, H529 to H538 and K531 to K540). (**B**) Principle of the assay. Human Plk1 is C-terminally fused to RLucII and Map is N-terminally fused to GFP10. When the RLucII and GFP10 moieties are brought closer than 10 nm, oxidation of the coelenterazine 400a substrate by RLucII induces an energy transfer from RLucII to GFP10, causing GFP10 to emit fluorescence. (**C**) The Plk1-Map interaction used in the assay strongly depends on pincer (H538 and K540) residues in the PBD of Plk1 required for its interactions with phosphorylated proteins. HEK293T cells were transiently transfected with a constant amount of Plk1-Luc plasmid and an increasing amount of Map-GFP plasmid. The BRET signal measured 48 hours later is plotted as a function of ratio GFP fluorescence/luciferase activity. Error bars: standard deviation of triplicate values from the same experiment. (**D**) Distribution of the screened compounds according to their effect on the Plk1-Map BRET signal. Left: distribution of the number of compounds according to the percentage of inhibition. Right: Percentage of inhibition values for each compound tested. A minimum threshold of 30% inhibition was applied as a first selection criterion.

To develop a cell-based assay for the identification of Plk1 interaction inhibitors, we used Bioluminescence Resonance Energy Transfer (BRET)^42^,^43^. The assay monitors the interaction in HEK293T cells between Plk1 and Map205, a protein that we previously identified as an interactor of Drosophila Polo (Plk1 ortholog) on its PBD^44^. Although no ortholog of Drosophila Map205 has been found in vertebrates, this protein robustly interacts with vertebrate Plk1. We chose this interaction because it readily occurs in interphase cells as it does not require priming phosphorylation by a mitotic kinase. Importantly, crystallography with zebrafish Plk1 has recently shown that Map205 nevertheless docks in the canonical phosphopeptide binding site of the PBD, with the natural phospho-mimetic Glu314 residue (Fig. 1A)^45^. In our assay, human Plk1 and a fragment of Drosophila Map205 (Map205_254-416_ = Map) are respectively fused to Renilla luciferase II (Luc) and GFP10 (GFP), giving rise to energy transfer when the proteins are in close proximity (Fig. 1B). Mutation of key residues in the PBD canonical phospho-binding site (H538A, K540M = Plk1^HK^) strongly decreases the BRET signal, demonstrating specificity for this site (Fig. 1C). All combinations of Plk1 and Map tagged with Luc and GFP were tested (Supplementary Fig. S1). The Plk1-Luc/GFP-Map combination was selected because it yielded the highest BRET dynamic window. We then generated clonal stable cell lines co-expressing these two fusion proteins. These cell lines behaved very similarly to each other in BRET, and we selected one line that gave a high and reproducible BRET signal, with a Z′ score of 0.78, indicative of a robust assay for the purpose of a chemical screen (Supplementary Fig. S1). Peptides derived from alternative Plk1 substrates were also tested instead of Map, but they did not yield a robust BRET signal (data not shown), as expected because they depend on mitotic phosphorylation for their interaction with Plk1.

### Chemical screen for PBD interaction inhibitors

We used our cell-based assay to screen a library of 112 983 compounds available in-house at IRIC’s High-Throughput Screening (HTS) platform (see Materials & Methods). Compounds were tested on cells in 384-well plates at concentrations between 10 μM and 25 μM, depending on their concentration in the original stocks solutions. BRET readings were taken 2 hours after treatment. Results obtained show a tight, close to normal distribution centered around zero inhibition relative to DMSO-treated controls (Fig. 1D). Compounds inducing at least 30% BRET inhibition were selected. From these, auto-fluorescent compounds and those that induced more than 90% reduction in RLucII luminescence were removed, leaving 800 primary hits, that we then re-tested using the same assay. In parallel, we tested these compounds in a control BRET assay for an unrelated pair of interacting proteins (BRAF-CRAF^46^) to exclude false-positive compounds that could reduce the BRET signal by interfering with light or by modifying the activity of GFP or Luc. We obtained 121 confirmed Plk1-specific BRET hits.

### Hit compounds interfere with mitosis

**Figure 2:**
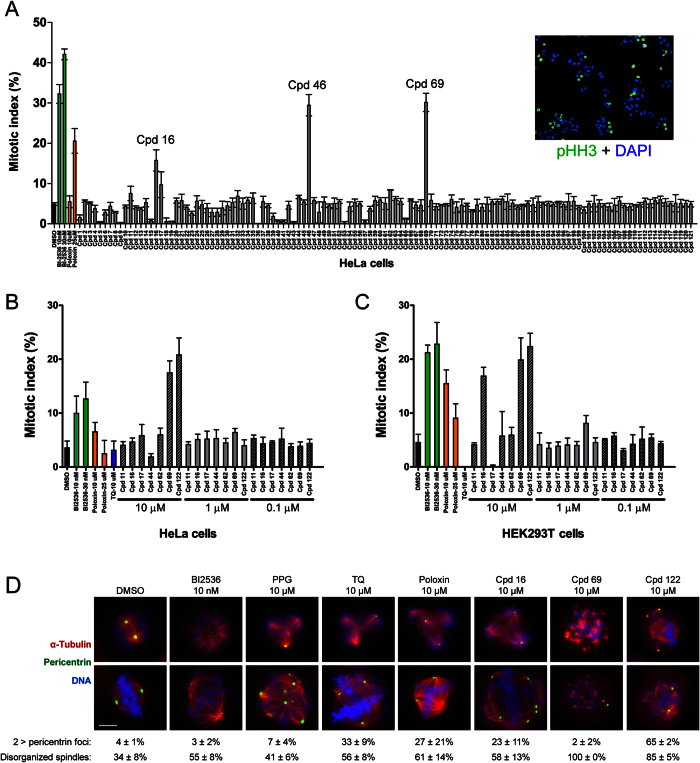
Some primary hit compounds increase the mitotic index and induce abnormal mitotic phenotypes. (**A**) The 121 primary BRET hits from the library were tested in a mitotic index assay (concentrations ranging from 10 μM to 25 μM). The known Plk1 inhibitors BI2536 and Poloxin were included at the indicated concentrations, for comparison. HeLa cells were treated for 7 hours before the immunofluorescence with phospho-Histone H3 antibody. Upper right: example of the image generated by the Operetta. Error bars: standard deviation of triplicate values from the same experiment. (**B**–**C**) Mitotic index for selected compounds purchased separately and re-tested at different concentrations in HeLa cells (**B**) and in HEK293T cells (**C**). (**D**) Plk1 inhibitors induce mitotic defects. HeLa cells were treated as above with the indicated compounds and examined by immunofluorescence to reveal α-Tubulin (red), the centrosome marker pericentrin (green) and DNA (DAPI, blue). The percentage of cells with more than 2 pericentrin foci and the percentage of cells showing a disorganized spindle are shown. Two representative images are shown for each treatment. For DMSO, the top image shows a normal disorganized spindle in prometaphase and the bottom image shows a normal metaphase. All experiments were done in triplicate and error bars correspond to standard deviation.

Plk1 is essential for mitotic progression. Therefore, if compounds completely block Plk1 functions, they should block cells in mitosis. We tested the effect of our 121 BRET hits on mitotic progression of HeLa cells by immunofluorescence against phospho-Histone H3 (pSer10), a well known mitotic epitope^47^. The percentage of cells positive for this marker (mitotic index) was scored. Only three hits, Cpd 16, Cpd 46 and Cpd 69 (Cpd: compound) markedly increased the mitotic index compared with DMSO-treated cells, suggesting that they inhibit Plk1 function enough to block or slow down mitosis at the concentration used (Fig. 2A). Cpd 46 and Cpd 69 are similar flavonoids, while Cpd 16 is distinct, with pyridine and benzyl groups separated by a sulfone and ester-containing linker (Supplementary Fig. S2). Under the conditions used, these 3 compounds at concentrations ranging between 10 μM and 25 μM induced a mitotic index around 20%, similar to Poloxin at 25 μM.

To ascertain that the effects on the mitotic index were attributable to compounds listed in our library, we purchased them independently from commercial vendors when possible. We obtained Cpds 16 and 69, but not Cpd 46. However, we were able to obtain a very similar analog, Cpd 122 in the same series (Supplementary Fig. S2). Cpd 122 was among our 800 primary hits and was a confirmed Plk1-specific BRET hit, but was not selected among our final list of 121 because it was marginally over the auto-fluorescence threshold. We also obtained other compounds that appeared to induce small increases in mitotic index (Cpds 11, 17, 44 and 62; Fig. 2A and data not shown). These molecules were tested at different concentrations on HeLa and HEK293T cells (Fig. 2B–C). At 10 μM, Cpds 69 and 122 induced a significant increase in mitotic index in both cell lines, while Cpd 16 was effective on HEK293T cells only at 10 μM. Little or no increase was observed at 1 μM (a small increase is seen for Cpd 69 in HEK293T cells) or 0.1 μM. Cpds 11, 17, 44 and 62 had no effect even at 10 μM.

We next examined the subcellular phenotypes of mitotic cells after treatment with Cpds 16, 69 and 122, which significantly increased the mitotic index. HeLa cells were treated with these compounds along with the known PBD inhibitors PPG, TQ, Poloxin and the KD inhibitor BI2536 as controls, and then stained against α-Tubulin, pericentrin (a centrosome marker) and DNA (Fig. 2D). In all cases, there was a marked increase in the frequency of disorganized spindles compared with DMSO-treated control cells. This is expected as Plk1 plays multiple roles in spindle assembly and maintenance. In addition, cells treated with Cpd 16, Cpd 122, TQ and Poloxin showed multiple foci of pericentrin. Cpd 69, an analog of compound 122, induced a similar phenotype at lower concentrations (data not shown). This phenotype was not seen after treatment with BI2536. These observations are consistent with previous results suggesting that PBD inhibition with Poloxin, more than KD inhibition, preferentially disrupts the ability of Plk1 to promote centrosome cohesion^35^. The same study suggested that this effect was due to interference with the role of Plk1 in promoting the activity of the centrosome protein Kizuna in preserving the cohesion of the pericentriolar material^48^. We did not explore further whether this mechanism was specifically disrupted.

### Biochemical validation of the ability of compounds to disrupt Plk1 interactions

**Figure 3:**
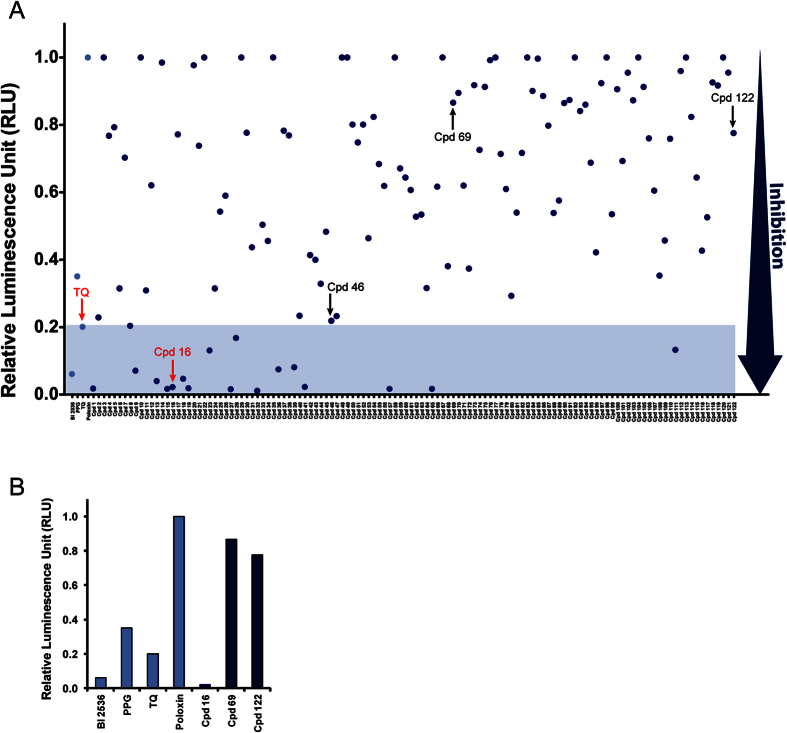
Some primary hit compounds interfere with the Plk1-Map interaction in a co-immunoprecipitation assay. (**A**) The 121 BRET hits (+Cpd 122) disrupt the Plk1/Map interaction to various degrees in this assay, some disrupting the interaction more efficiently than TQ (blue zone), including Cpd 16. For each compound, values (relative luminescence unit) were calculated relative to the maximal value in each column of compounds (see Table S1 for raw data and analysis). (**B**) Focus on selected hit compounds and controls. Note that like TQ and Cpd 16, BI2536 also disrupts the interaction in this assay. All values are averages of duplicates from the same experiment.

Compounds that decrease the BRET signal are predicted to disrupt the Plk1-Map interaction. However, they could act without disrupting the interaction, for example by changing the conformation of one of the BRET partners and thus the efficiency of the energy transfer. To test which compounds really disrupt the Plk1-Map interaction, we used a co-precipitation assay based on the Luminescence-based mammalian interactome mapping (LUMIER) assay^49^, testing the 121 hits of our primary screen (+Cpd 122). HEK293T cells stably expressing Map-Luc/GFP-Plk1 were treated for 2 hours with compounds (between 10 μM and 25 μM) in 96-well plates. After cell lysis, GFP-Plk1 was immunoprecipitated and co-purified Map-Luc was measured by luminescence following addition of the luciferase substrate, coelenterazine. Our 121 BRET hits disrupt the Plk1/Map_254-416_ interaction to various degrees in this assay (Fig. 3A and Supplementary Table S1). We focused our attention on the compounds that also induced an increase in mitotic index, in addition to scoring as BRET inhibitors. Cpd 16 disrupts the interaction with similar or better efficiency than TQ or PPG (Fig. 3A-B). Surprisingly, Cpds 69 and 122 have no effect on the Plk1-Map interaction in this assay. The KD inhibitor BI2536 strongly inhibited the Plk1-Map interaction (Fig. 3A-B). We previously observed the disruption of the Plk1-Map205 interaction by BI2536. It can be explained by a steric clash between KD-bound BI2536 and the PBD, which in turn destabilizes the interaction with Map205^50^. Thus, compounds that interfere with the Plk1-Map interaction can act allosterically on Plk1. Compounds that disrupt the interaction both in BRET and in co-IP but have no effect on the mitotic index could possibly still inhibit the PBD although not efficiently enough to cause problems in mitosis. They could also inhibit the Plk1-Map interaction by binding to Map instead of Plk1 or by a non-specific effect in the cell. Considering all variables, Cpd 16 clearly appeared as our most promising hit as it was the only compound clearly active in BRET, mitotic index and Plk1-Map co-IP assays (Supplementary Table S2). We therefore sought to investigate its mode of action through the use of *in vitro* assays.

**Figure 4:**
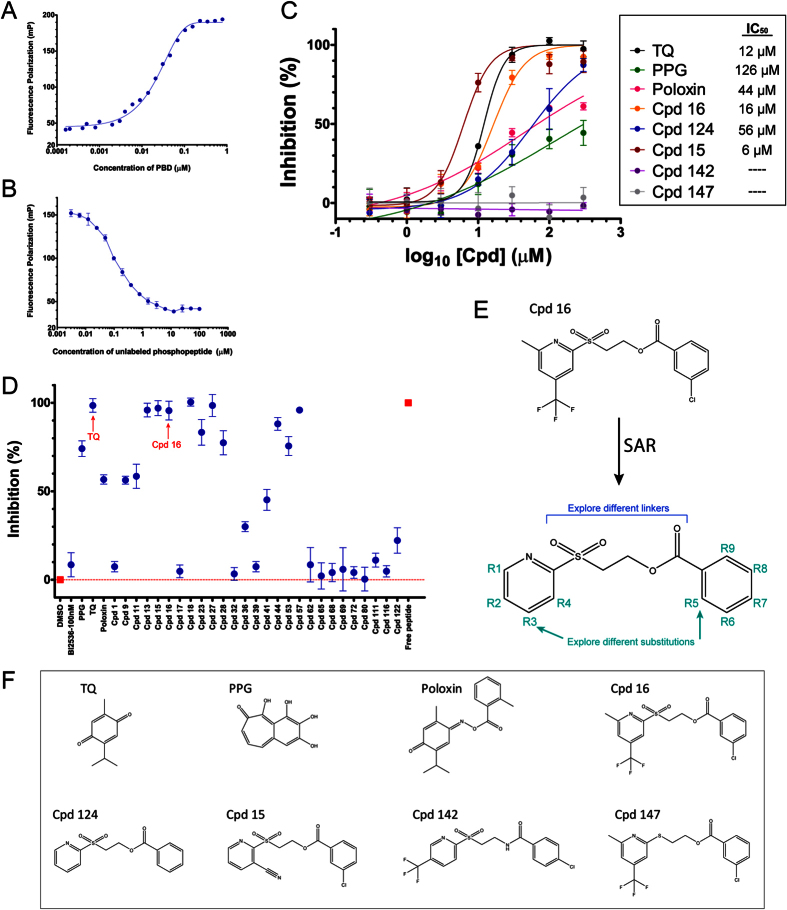
Some of the compounds identified interfere with the interaction between the PBD and binding peptides in a Fluorescence Polarization (FP) assay. (**A**) The FP signal increases when a FITC-labeled PBD-binding phosphopeptide (10 nM) is incubated with increasing concentrations of GST-PBD_367-603_. (**B**) Competitive displacement of the FITC-phosphopeptide (10 nM) from the GST-PBD_367-603_ (used at EC_65_ = 38 nM) by the corresponding unlabeled phosphopeptide added at increasing concentrations. Experiment was done in duplicate. Error bars: range between values. (**C**) Inhibition curves of compounds assayed by FP. GST-PBD_367-603_ (38 nM) was pre-incubated for 1 hour with indicated compounds before addition of the FITC-phosphopeptide. TQ and Cpd 16 show similar IC_50_ values. Experiment was done in triplicate. Error bars: standard deviation. (**D**) Targeted screen of selected compounds in the FP assay. GST-PBD_367-603_ (38 nM) was pre-incubated for 1 hour with different compounds at 50 μM before addition of the FITC-phosphopeptide. Values are means of triplicates from the same experiment (±standard deviation). (**E**) Summary of SAR strategy targeting substitutions on the cycles and the linker between them. (**F**) Chemical structures of published PBD inhibitors used in this study, Cpd 16 and 4 selected analogs of Cpd 16 tested in FP in panel C.

To test if compounds interfere with the ability of Plk1 to interact with its substrates by its canonical site on the PBD, we used a fluorescence polarization (FP) assay developed by Reindl *et al*., by which they identified TQ and Poloxin as PBD inhibitors^34^,^51^. In this assay, an optimal phosphopeptide for PBD binding is coupled with a FITC fluorophore and its binding to purified recombinant PBD of Plk1 is monitored by the change in polarization of incident light. The FP signal increased to a maximum with increasing concentrations of PBD (Fig. 4A) and decreased when the FITC-labeled peptide was competed off with an equivalent unlabeled peptide (Fig. 4B). Cpd 16 was active in this assay, with an IC_50_ around 16 μM, which is similar to that obtained with TQ (around 12 μM) and better than Poloxin (around 44 μM) (Fig. 4C). The peptide used in this assay is completely different from Map and the only other component in the reaction that is in common with the BRET and co-IP assays is the PBD. Therefore, the FP results strongly suggest that Cpd 16 interferes with the PBD itself.

We also set up similar FP assays monitoring the interaction of the PBD or full-length Plk1 with a small peptide derived from Map205 and coupled to FITC (Supplementary Fig. S3). We observed that many of our compounds, including Cpd 16, TQ, Poloxin and PPG, disrupted the PBD-Map interaction in the FP assay, further reinforcing the conclusion that these compounds act on the PBD (Supplementary Fig. S3). However, the affinity between whole Plk1 and the Map peptide was seemingly very high and we did not observe much impact of our compounds even at 100 μM in this assay (Supplementary Fig. S3). This result may be explained by the known function of the KD of Plk1 in an interdomain interaction that stabilizes the conformation of the PBD which is capable of a very stable interaction with Map205^45^. This effect may be less dominant in cell-based assays such as BRET, because in a cellular environment, Plk1 exists in equilibrium between different forms where the interdomain interaction is modulated by post-translational modifications and protein interactions^33^.

We selected several additional compounds to be tested in the PBD-phosphopeptide FP assay, based on their activity in BRET, mitotic index and co-IP assays (Fig. 4D and Supplementary Table S2). Cpds 69 and 122 showed little or no activity in the FP assay, as predicted since they are inactive in co-IP. Interestingly, several additional compounds were active in FP, but because none of them significantly perturbed mitosis, we did not prioritize them for further analysis.

### Cpd 16 is unstable and generates an active vinyl sulfone

With the goal to increase the potency of Cpd 16, and to obtain hints regarding its mechanism of action, we initiated a Structure-Activity Relationship (SAR) analysis (Fig. 4E). We obtained commercially available analogs of Cpd 16 and we also prepared some derivatives using well established synthetic methods (Supplementary Fig. S4). The SAR with the FP assay revealed that the sulfone and the ester functionalities are crucial to maintain good activity. The substitution patterns on both aromatic rings do not have a strong effect on the potency. However, activity was abolished by replacement of the sulfone by a sulfoxide (Cpd 148) or a thioether (Cpd 147), or by the replacement of the ester by an amide functionality (Cpd 142) (Fig. 4C, F and Supplementary Table S3).

**Figure 5:**
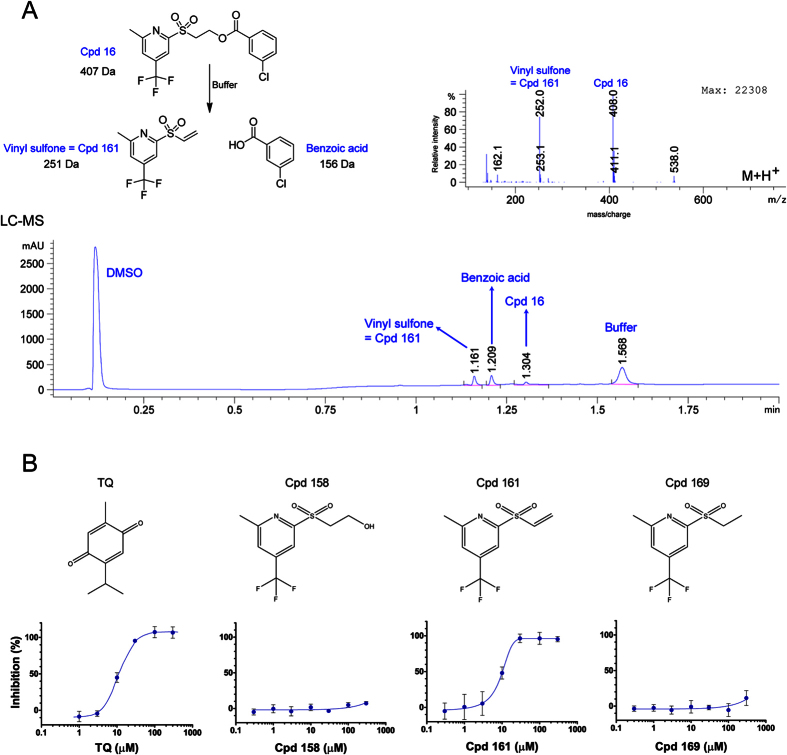
Cpd 16 spontaneously fragments to generate a vinyl sulfone whose vinyl group is required for PBD inhibition. (**A**) Cpd 16 fragments into vinyl sulfone and benzoic acid products in an aqueous solution at approximate pH 8.0. Upper left: Diagram showing the reagent and products. Bottom: Diode array detector chromatogram (DAD) showing all the components detected after approximately 10 minutes in FP buffer. Upper right: Vinyl sulfone was detected on the mass spectrometer (m + 1 = 252) by LC-MS. (**B**) Inhibition dose-response curves obtained by FP with the chemical structures of the compounds tested. GST-PBD_367-603_ at 38 nM (EC_65_) was incubated for 1 hour with the compound before addition of the FITC-phosphopeptide (10 nM). Experiment was done in triplicate. Error bars: standard deviation.

Based on these observations, we speculated that the sulfone undergoes a β-elimination of the benzoate subunit to generate a vinyl sulfone which is responsible for the biological activity of this class of compounds. Using LCMS, we evaluated the stability of Cpd 16 in the buffer used in the FP assay. After a few minutes of incubation in that buffer, LC-MS revealed that fragmentation occurred and the vinyl sulfone (Cpd 161) together with the benzoic acid are the major components of the solution (Fig. 5A). The sulfone β-elimination is a base-catalysed process and the buffer used in the assay is basic enough to allow the reaction to occur. Under neutral or acidic conditions, the compound was very stable and no fragmentation was observed. The vinyl sulfone (Cpd 161) was easily prepared from the alcohol (Cpd 158) (Supplementary Fig. S4) and showed the same activity as Cpd 16 in the FP assay. As expected, saturation of the vinyl group completely abrogated activity in FP (Cpd 158 and Cpd 169; Fig. 5B).

### The vinyl sulfone is a protein alkylator and is required for PBD inhibition

**Figure 6:**
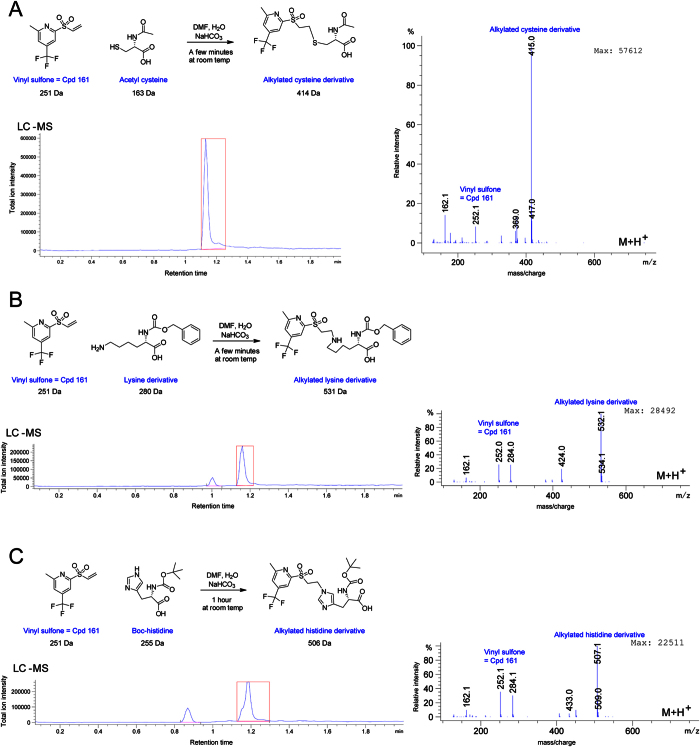
Cpd 161 alkylates nucleophilic amino acid side chains. Alkylated cysteine (**A**), lysine (**B**) and histidine (**C**) derivatives were detected by LC-MS. For the 3 amino-protected amino acids, the scheme of the reaction, Total Ion Chromatogram (TIC), and mass spectrum (m + 1) are presented. Cpd 161 reacted to completion with amino-protected cysteine (**A**) and lysine (**B**) after approximately 10 minutes at room temperature. The reaction also occurred with amino-protected histidine (**C**) but was slower. TIC peaks corresponding to reaction products analyzed by MS are indicated by red boxes.

**Figure 7:**
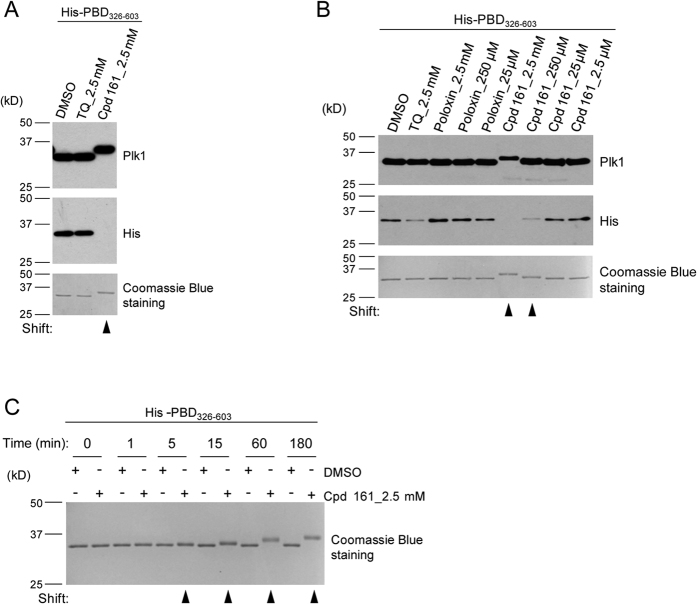
Covalent reaction of Cpd 161 with the PBD is detected by SDS-PAGE. (**A**) Alkylation of the PBD by Cpd 161 is detected as a shift in apparent molecular mass. His-PBD_326-603_ (2 μg) was treated for 4 hours with DMSO, TQ (2.5 mM) or Cpd 161 (2.5 mM). Fractions of the reaction equivalent to 0.1 μg of protein per lane were subjected to Western blot analyses using antibodies directed against Plk1 or the poly-histidine tag. Fractions of the reaction equivalent to 1 μg were loaded on a gel for Coomassie Blue gel staining. (**B**) Alkylation of the PBD by Cpd 161 is dose-dependent. His-PBD_326-603_ (2 μg) was treated for 3 hours with different concentrations of Cpd 161, Poloxin and TQ. Note that only Cpd 161 can induce a clear shift of the protein, which is dose-dependent. Western blotting and Coomassie Blue gel staining were performed as described in (**A**). (**C**) Alkylation of the PBD by Cpd 161 is time-dependent. His-PBD_326-603_ (2 μg) was treated with DMSO or 2.5 mM of Cpd 161 for different times before SDS-PAGE analysis. Cropped images of blots and gels are shown; uncropped images are provided in Supplementary Fig. S5.

Vinyl sulfones are reactive species that can alkylate substrates as a result of a nucleophilic attack on the distal carbon of the vinyl group. To evaluate the potential of Cpd 161 (vinyl sulfone) to react with proteins, we tested its reaction with nucleophilic amino acid side chains groups. After incubation for only a few minutes at room temperature, Cpd 161 reacted to completion with N-terminally protected lysine or cysteine. It also reacted with histidine, but the reaction was slower (Fig. 6). These results indicate that Cpd 161 is a potent alkylator of the nucleophilic lysine, cysteine and histidine side chains. To test whether Cpd 161 could react covalently with the PBD, we incubated them together and examined the molecular mass of the protein by SDS-PAGE (Fig. 7). When revealed by Coomassie Blue staining of the gel or by Western blot with a Plk1 antibody directed against the PBD, the PBD appeared higher in the gel, a few kDa above the protein treated with DMSO alone (Fig. 7A). As the mass of Cpd 161 is 251 Da, this shift suggests that several units of Cpd 161 can react with one unit of PBD. Consistent with this idea, the extent of the shift depended on the concentration of Cpd 161 used (Fig. 7B) and also on the incubation time (Fig. 7C). Interestingly, recognition of the protein by an antibody directed against the poly-histidine tag, which is present at the N-terminal of the recombinant PBD, was reduced or even lost after reaction between the PBD and Cpd 161, probably because alkylation of histidine residues in the tag prevents recognition by the antibody (Fig. 7A-B). These results reinforce the conclusion that Cpd 161 inhibits PBD interactions by alkylation.

### Thymoquinone and Poloxin are also protein alkylators

**Figure 8:**
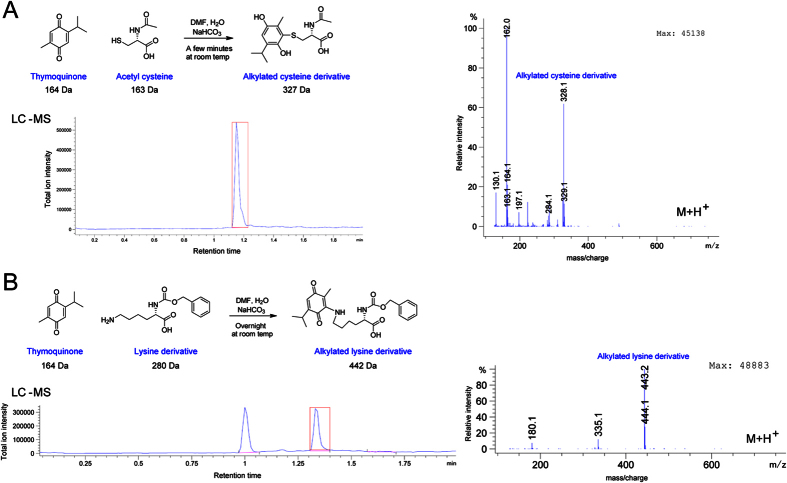
TQ alkylates nucleophilic amino acid side chains. Alkylated cysteine (**A**) and lysine (**B**) derivatives were detected by LC-MS. For the 2 amino-protected amino acids, the scheme of the reaction, Total Ion Chromatogram (TIC), and mass spectrum (m + 1) are presented. TQ reacted to completion with amino-protected cysteine (**A**) and lysine (**B**). With cysteine (**A**) completion occurred after only approximately 10 minutes at room temperature and the reaction was much slower (several hours) with lysine (**B**). TIC peaks corresponding to reaction products analyzed by MS are indicated by red boxes. No reaction was detected with amino-protected histidine (data not shown).

**Figure 9:**
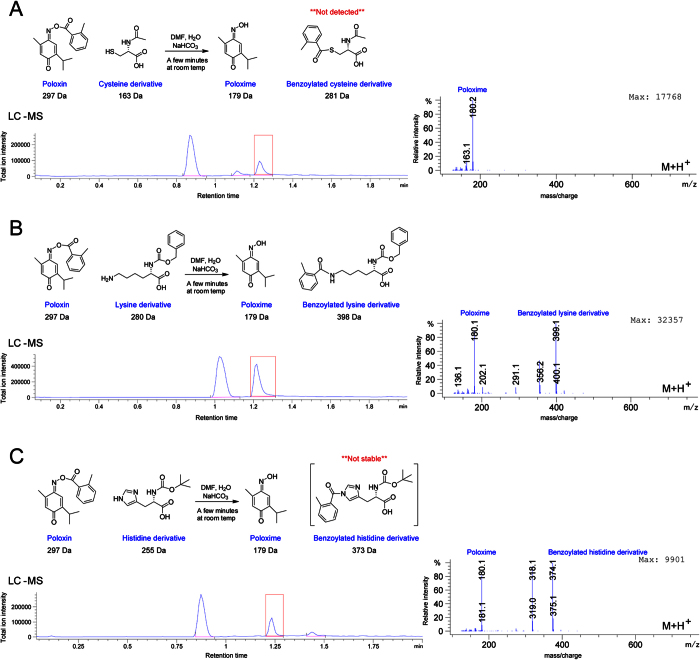
Poloxin alkylates nucleophilic amino acid side chains. Benzoylated lysine (**B**) and histidine (**C**) derivatives were detected by LC-MS. For the 3 amino-protected amino acids, the scheme of the reaction, Total Ion Chromatogram (TIC), and mass spectrum (m + 1) are presented. Poloxin did not react with protected cysteine (**A**); only the hydrolysed Poloxime form was detected. Poloxin reacted to completion with amino-protected lysine (**B**) and histidine (**C**) after only approximately 10 minutes at room temperature, where a cleaved Poloxime form was also observed in all cases. TIC peaks corresponding to reaction products analyzed by MS are indicated by red boxes. Note that benzoylated histidine derivative was not stable and was no longer detected after a few hours, when only the Poloxime form was detected (data not shown).

Thymoquinone and Poloxin were the first PBD inhibitors identified and arguably the most potent small molecule PBD inhibitors in cells. It was proposed that these molecules inhibit the binding of Plk1 interaction substrates by competing with the same site on the PBD^34^,^36^. Because TQ behaved very similarly to Cpd 161 in all our cell-based and biochemical assays so far, we decided to test if it could also act as an alkylator. Interestingly, TQ contains a double bond whose electrophilic potential is predicted to be enhanced by the presence of a neighboring carbonyl. Strikingly, TQ reacted to completion with amino protected cysteine in only a few minutes at room temperature (Fig. 8A). We could detect the reaction product with as little as 50 μM TQ that reacted at neutral pH (PBS) with acetyl cysteine; we could not test lower concentrations because of the detection limit of the liquid chromatography (data not shown). TQ also reacted with protected lysine, although the reaction was slower (Fig. 8B). No reaction was observed with histidine (data not shown). For Poloxin, in the presence of protected cysteine (Fig. 9A) only the hydrolysed Poloxime (Thymoquinone-oxime) form was detected. Poloxin also reacted to completion with amino-protected lysine and histidine within only a few minutes at room temperature (Fig. 9B,C). Although Poloxin contains the chemical function that reacted in TQ, its reaction with lysine and histidine occurred by a distinct mechanism leading to the formation of benzoylated amino acid derivatives. However, the benzoylated histidine derivative was not stable, and only the hydrolysed Poloxime form was detected after a few hours (data not shown).

**Figure 10:**
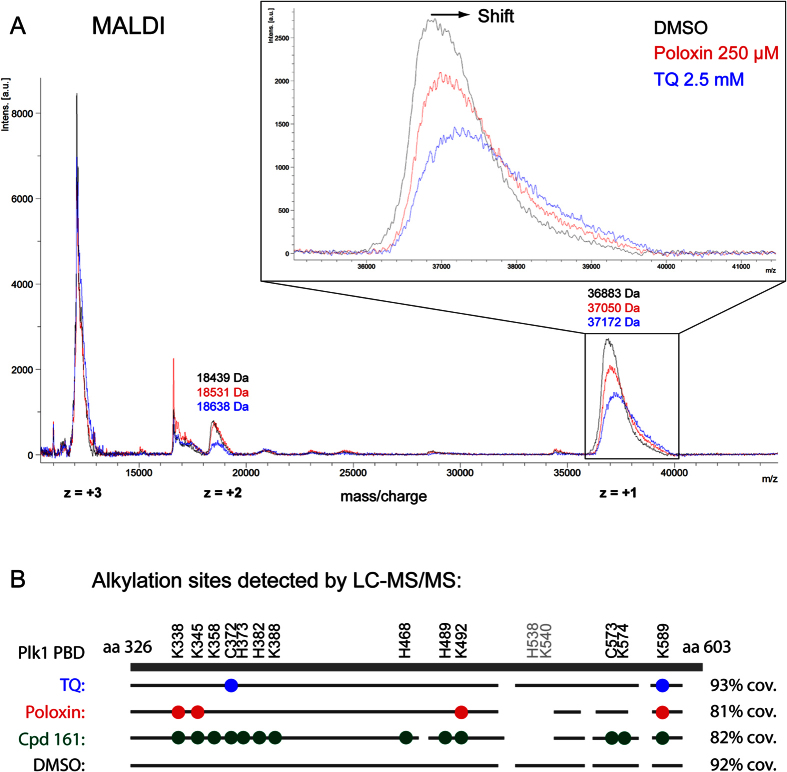
Covalent reaction of TQ and Poloxin with the PBD is detected by mass spectrometry. (**A**) His-PBD_326-603_ (4 μg) was treated with 2.5 mM TQ, 250 μM Poloxin (2.5 mM precipitated), or DMSO (negative control) for approximately 2 hours. Samples were submitted to MALDI-TOF analysis. The full spectra showing the 3 forms of the protein (+1 peak around 36.9 kDa, +2 peak around 18.5 kDa, and the +3 peak around 12 kDa), are shown. The +1 form was zoomed in to better evaluate the peak displacement of the PBD caused by alkylation by TQ and Poloxin. (**B**) Alkylation sites detected by LC-MS/MS on tryptic digests of alkylated PBD. Residues observed to be alkylated by TQ, Poloxin and Cpd 161 are indicated by colored circles. In each case, the grey line indicates the sequence covered by the peptides observed in the analysis, with percentages of PBD sequence on the right.

Although TQ and Poloxin are capable of alkylating amino acid side chains, they did not cause marked mobility shifts in a gel like Cpd 161 (Fig. 7A-B). We turned to Matrix-Assisted Laser Desorption/Ionisation – Time of Flight (MALDI-TOF) as a more sensitive method to test if these compounds could react covalently with the PBD. Analysis of the DMSO-treated PBD revealed a signal with a maximum intensity around 36 883 m/z, corresponding to the protein bearing a single positive charge. After incubation of the protein with TQ or Poloxin, the protein signals reproducibly shifted to higher m/z values, with a maximum at approximately 37 000–38 000 depending on the experiment (Fig. 10A), suggesting a covalent reaction with approximately one or two units of each compound on average under the conditions used. A weaker signal was observed around 18 439 m/z, corresponding to the protein bearing a double positive charge. This signal was also shifted up by treatment with TQ or Poloxin. These results confirm that TQ and Poloxin react covalently with the PBD, as suggested by the observed reactivity with nucleophilic amino acid side chains. We analysed the protein by MALDI-TOF after reaction with Cpd 161, but the protein signal reproducibly disappeared for unknown reasons. It may be that the incorporation of multiple units of Cpd 161 in the protein changes dramatically its ionization and/or desorption properties in the sample. We also tried mass spectrometry using electrospray on the whole PBD, but the protein fragmented in the analysis (data not shown).

To map alkylation sites on the PBD, we turned to LC-MS/MS analysis of alkylated proteins after digestion with trypsin (Fig. 10B). We detected alkylation by TQ, Poloxin and Cpd 161, with profiles consistent with the chemical reactivity observed above. While addition of TQ was found on cysteine and lysine residues, addition of Poloxin was detected on multiple lysine residues, and Cpd 161 alkylated cysteines, lysines and histidines. For all 3 compounds, alkylated residues were detected outside the PBD substrate binding pocket (discussed below). We conclude that Cpd 161, TQ and Poloxin are non-specific protein alkylating agents.

## Discussion

Our goal was to identify new inhibitors of Plk1 interactions that would be more specific to Plk1 and active *in vivo*. To this end, we designed the first cell-based screening system that monitors the capacity of Plk1 to interact with a target via its PBD. We used this assay to conduct a HTS chemical screen. After characterization in secondary assays, we found that our only potent, on-target hit with a disruptive effect on mitosis (Cpd 161) is an alkylator and that its inhibition of the PBD depends on its alkylating function. Moreover, we showed that TQ and Poloxin, compounds that provided the first proof of concept for PBD inhibition by non-peptide small molecules are also alkylators of nucleophilic amino acid side chains. The potential of Poloxin to act as an alkylator on the PBD was proposed based on SAR in a recent study, but protein alkylation by Poloxin was not demonstrated^52^.

As Cpd 16 rapidly fragments to generate a vinyl sulfone that is capable of alkylating lysine, cysteine and histidine side chains by reacting with their strongly nucleophilic nitrogen and sulfur atoms, it is probably capable of alkylating any protein that contains such residues. It is therefore impossible to conclude that the observed effect of Cpd 16 on mitosis is due to its effect on Plk1. It could be due to interference with any important mitotic regulators, microtubules or even chromosomes, or a combination of several off-targets. In this regard, it is worth noting that treatment of cells with Cpd 16 did not result in a significant decrease in localization of Plk1-GFP or endogenous Plk1 from mitotic centrosomes, kinetochores or the cytokinetic midbody, as predicted for a PBD inhibitor that would be effective in cells (data not shown). Similar conclusions can be reached for TQ and Poloxin: they are also non-specific alkylators and the effects they have on mitosis and cell viability could easily be due to off-target effects^34^,^35^. A decrease in Plk1 localization to centrosomes was reported in the presence of TQ or Poloxin, but we failed to reproduce this result (data not shown). Although we added up to 100 μM of each compound to the medium, it is impossible to know the concentrations reached inside the cell. An intracellular concentration of a compound that is sufficient to kill the cell due to unspecific alkylations may be insufficient to disrupt Plk1 localization.

Our FP results show that Cpd 161 inhibits PBD binding activity. Although Cpd 161 is in principle capable of alkylation on any exposed lysine, histidine and cysteine side chains, such modifications are not predicted to result in PBD inhibition unless they disrupt its binding site. Such inhibition could occur by alkylation directly obtruding the binding site or by alkylation at a distant site that would induce a conformational change allosterically disturbing the binding site. We used molecular docking calculations to probe for the putative interaction site(s) and mode of binding of Cpd 161 on the PBD structure^53^. Calculations were made in a non-biased manner, using the whole PBD as a possible binding space. Results are shown in Supplementary Fig. S6 where the two most probable binding modes are shown. Interestingly, both predictions showed Cpd 161 bound precisely within the canonical binding site of the PBD. There, His538 and Lys540, the “pincer” residues that are essential for contact with a phospho-serine or phospho-threonine are exposed and could possibly react with the vinyl group in Cpd 161. Our chemical results would be also consistent with the reaction of Lys540 with TQ or Poloxin. Alkylation at His538 or Lys540 would cause steric hindrance and block protein interactions with the PBD in its binding pocket. Alkylation at these residues was not detected in our LC-MS/MS analysis (Fig. 10B). However, if His538 or Lys540 were alkylated, the trypsin may not be able to cleave after Lys540, and the resulting tryptic peptide would be very long (38 residues) and thus difficult to detect. Interestingly, we saw that non-alkylated peptides covering His538 and Lys540 were not detected in PBD treated with Poloxin and Cpd 161, possibly because the protein was heavily alkylated at this region, while non-alkylated peptides covering this region were detected in PBD that reacted with TQ and DMSO alone.

Interestingly, alkylation of two residues was found very near the phosphopeptide binding site in the PBD and could contribute to blocking interactions: His489 for Cpd 161 and Lys492 for Cpd 161 and Poloxin^53^. Lys492 was previously shown to be mono-ubiquitylated in a mechanism that allows the release of Plk1 from its docking partners at kinetochores in metaphase^54^. It is therefore possible that alkylation at this site by Cpd 161 or Poloxin contributes to impede on the ability of the PBD to engage in protein interactions. It has been proposed, based on theoretical calculations, that TQ and Poloxin could potentially alkylate Cys544 after binding the PBD at its canonical site^55^. However, we did not observe alkylation at this site and Poloxin did not react with acetyl cysteine. Potent inhibitors that act by site-specific alkylation of nucleophilic amino acid side chains have been identified^56^,^57^,^58^. These tend to involve a basal non-covalent interaction at the site of alkylation that can be observed even for analogs that are incapable of alkylation. For example, an irreversible, covalent inhibitor of CDK2 was developed by the addition of a vinyl sulfone function to a pre-existing non-covalent inhibitor^57^. As analogs of Cpd 161 that lack the vinyl group are inactive in PBD inhibition up to at least 300 μM, it is rather unlikely that Cpd 161 could be a good starting scaffold to develop any useful PBD inhibitor.

Non-specific alkylators such as Cpd 161, TQ and Poloxin are reminiscent of Pan-Assay Interference Compounds (PAINS) which are infamous to medicinal chemists because they stumble on them so often as non-specific hits in screening assays^59^. It is estimated that approximately 7% of compounds in IRIC’s chemical library may be considered as PAINS, which is low compared to other chemical libraries (unpublished observations). Of such PAINS, non-specific alkylators constitute only a small fraction. When identified, problematic compounds in a library should be flagged.

We hope that the assay we have developed will be used to screen additional libraries and that this will lead to the identification of more promising lead compounds with PBD inhibitor activities. Our assay is the first to function in cells for this purpose. This is an advantage because it allows the identification of membrane permeable and biologically active compounds right away, unlike *in vitro* assays using the PBD in solution. Our BRET-based assay was enabled by the fact that the Plk1-Map interaction occurs in interphase and not only in mitosis like most Plk1-substrate interactions. The high affinity of the Plk1-Map interaction favors a high stringency in screens, and thus inhibitors identified are likely to be potent. However, the Plk1-Map affinity could be too high to pick up lower-affinity compounds that could be improved later through SAR. Nevertheless, we know that in the cell, Plk1 and Map205 are dynamic, their interaction being disrupted by phosphorylation of both proteins^44^,^50^, which likely contributes to the capacity of the assay to identify inhibitors. In potential future use, the assay could benefit from variations in Plk1-Map affinity by mutations in the Map peptide utilized. Like *in vitro* assays, our cell-based assay also has the potential to produce false positives hits that can affect the interaction indirectly. The higher complexity of a cell-based system actually increases this risk. For this reason, the secondary assays using mitotic index, co-IP and FP that we have put in place are essential to determine which compounds act on target. In future screens, a special effort should be made to use more diverse libraries enriched with compounds of higher molecular weight and bearing more complex three-dimensional topologies. This is a crucial concern for the identification of protein/protein interaction inhibitors because most protein interactions occur via a larger surface than enzymatic active sites targeted in traditional screens^60^.

## Materials and Methods

### Plasmids

cDNAs coding for the RLucII and GFP10 tags were introduced in the pIRESHyg3 plasmid, where cDNAs for the human full length Plk1 and a fragment of Drosophila Map205 (Microtubule-associated protein 205) from amino acid residues 254 to 416 (Map205_254-416_ = Map) were then introduced in C or N-term to obtain 8 constructs. The 8 pairs of plasmids were transiently transfected and tested in BRET 48 hours later to select the better combination. A pIRESPuro3 plasmid, where we introduced GFP10 and Map, was also created to generate the clonal stable cell line used in the screen. The Plk1-PBD^HK^ (H538A and K540M) mutant was generated using QuikChange^®^ Lightning Site-Directed Mutagenesis Kit (Agilent) following the manufacturer’s protocol.

### Cell culture, transfections and cell lines

HEK293T cells were cultured in DMEM medium (Invitrogen) supplemented with 10% FBS (Invitrogen) and with penicillin/streptomycin (Wisent). Transfections were done using FuGENE (Promega). For the screen, a HEK293T clonal stable cell line expressing Plk1-Luc and GFP-Map was generated. The pIRESHyg3-Plk1-RLucII plasmid was first transfected in HEK293T cells and selection with hygromycin (30 μg/ml) was applied before isolating clones. Transient transfection of the pIRESPuro3-GFP10-Map plasmid was done in a few clones expressing Plk1-Luc to test BRET. One of these clones was selected to be stably transfected with the pIRESPuro3-GFP10-Map plasmid. After selection with puromycin (0.3 μg/ml) and clone isolation, expression levels of GFP and the Luc fusion proteins and the BRET ratio were measured to select the stable cell line to use for the chemical screen. For titration curves, 15 000 HEK293T cells were seeded in 96-well white plate (CulturePlate; PerkinElmer Inc.) coated with poly-L-ornithine and transfected the day after. The amount of transfected RLucII plasmids was keep constant (40 ng), while the amount of GFP10 plasmid was increased (between 0 and 400 ng). The empty pIRESHyg3 was used to complete to 440 ng of DNA transfected per well where needed. FuGENE was used in a ratio 3:2 (3 μL FuGENE: 2 μg DNA). Transfections for titration curves were done in triplicate.

### BRET

For BRET on transiently transfected cells, 48 hours after the transfection, medium was removed, cells were washed twice with PBS, and then Tyrode solution (140 mM NaCl, 2.7 mM KCl, 12 mM NaHCO_3_, 5.6 mM D-glucose, 0.49 mM MgCl_2_, 0.37 mM NaH_2_PO_4_, 25 mM HEPES, pH 7.4, where 1 mM CaCl_2_ was freshly added) was added to the cells. Total fluorescence was first measured using a FlexStation II microplate reader (Molecular Devices), where the GFP10 was excited at 400 nm and read at 510 nm. Then, cells were treated for 5 minutes with 2.5 μM coelenterazine 400a (Gold Technology) before reading BRET2 using the Mithras LB940 microplate reader (Berthold Technologies). BRET2 signal corresponds to the light emitted by the GFP10 acceptor constructs (515 nm ± 20 nm) divided by the light emitted by the RlucII donor constructs (400 nm ± 70 nm). Net BRET2 was calculated by subtracting the background BRET ratio of the RLucII plasmid alone from the BRET ratio obtained by the two BRET partners. The Net BRET2 was plotted according to the GFP10/RLucII ratio, which was obtained by dividing the total fluorescence by the RLucII emission.

For the high throughput chemical screen, cells were amplified and frozen as multiple 1 ml aliquots containing 25 millions of stable HEK293T Plk1-Luc/GFP-Map cells to ensure that cells at the same passage number are used during the screen. HEK293T Plk1-Luc/GFP-Map cells were seeded at 12.5 millions per double CellStack (Corning). Five days later, cells were counted and re-suspended in Tyrode solution. 50,000 cells per well (45 μl/well) were seeded in 384-well white plates (Greiner Bio One). Library compounds were added (0.25 μl) to the cells (between 10–25 μM final concentration) using the 384 pin tool head of a Biomek liquid handler (V&P Scientific), and plates were incubated for 2 hours at 37 °C in a CytoMat automated incubator. Total GFP10 fluorescence was read using an EnVision plate reader (Perkin Elmer inc.) with an excitation filter for 400 nm ± 20 nm and an emission filter for 510 ± 20 nm) Coelenterazine 400a solution containing 1% Pluronic (to avoid precipitation) was added (2.5 μM final) to the cell and plates were incubated for 10 minutes before the BRET2 measurements using a SpectramaxL luminometer (Molecular Devices) with an excitation filter for 400 nm ± 10 nm and an emission filter for 510 ± 10 nm). The Z-factor was calculated using the equation: 1 − (3(σ_p_ + σ_n_)/|μ_p_ − μ_n_|), where σ correspond to the standard deviation and μ to the mean of positive (p) or negative (n) controls^61^. BRET inhibition was calculated with the formula: 100 − [(BRET_sample_\BRET_DMSO_) × 100].

For the confirmation screen, the same procedure as for the primary screen was followed, except that HEK293T cells were also seeded at 6.25 millions of cells per simple CellStack on day 1 for transient transfections. These cells were transfected on day 3 with Luc-Map (5 μg) and Plk1-GFP (25 μg) pIRESHyg3 plasmids using polyethylenimine (PEI). To identify false-positives compounds, Luc-CRAF (4 μg) and GFP-BRAF (20 μg) plasmids were also transfected with PEI following the published procedure^46^.

Note that BRET1 (Mithras LB940 microplate reader equipped with acceptor (530 ± 20 nm) and donor (480 ± 20 nm) filters) was used at the beginning of the study due to the lack of the BRET2 filters. BRET2 probes (RlucII and GFP10) can be read with either BRET1 or BRET2 filter sets^62^ and the main difference is that the BRET values are higher when using the BRET1 filters.

### Immunofluorescence and microscopy

For the mitotic index assay, 4000 HeLa cells or 5000 HEK293T cells per well were seeded in a 96-well plate. The day after, cells were incubated for 7 hours with different compounds, and then fixed by adding formaldehyde (4% final concentration) directly to each well. Plates were incubated 20 minutes at 37 °C, and then wells were rinsed three times with TBS-Tween 0.1%. A 1 hour block with PHEM buffer (60 mM Pipes, 25 mM HEPES, 10 mM EGTA, 4 mM MgSO_4_, pH 6.9) containing 2% BSA and 0.1% triton was performed, before addition of the primary antibody to the wells. The anti-phospho-Histone H3 (Ser10) antibody (EMD Millipore) was diluted 1:300 in PHEM buffer containing 2% BSA and added to the wells for 2 hours at room temperature in the dark. Wells were then washed three times with TBS-Tween 0.1%, before addition of the secondary antibody Alexa Fluor 488 anti-rabbit (Invitrogen) diluted 1:200 in PHEM buffer containing 2% BSA. Plates were incubated for 1h 30 at room temperature in the dark, and then wells were washed 3 times with TBS-Tween 0.1%. A solution of Mowiol containing DAPI was finally added to the wells. Plates were kept at 4 °C in the dark until imaging using the 20x objective on an Operetta system (Perkin Elmer inc.). The mitotic index, corresponding to the percentage of pHH3 positive cells relative to the total number of DAPI-stained cells detected was calculated by the Operetta using the Harmony 3.5 software. Experiments were done in triplicates.

For the mitotic defect phenotypes, 25 200 HeLa cells were plated on poly-L-lysine coated round cover slips in 24-well plates. The day after, cells were treated for 7 hours, and then washed with PHEM buffer at 37 °C before fixation in PHEM buffer containing 4% formaldehyde for 20 minutes at 37 °C. Cells were washed three times with TBS-tween 0.1% and plate was kept overnight at 4 °C. Next steps were followed as previously described except that coverslips were incubated in a dark wet chamber with antibodies. Anti-pericentrin (diluted 1:1000; Abcam), anti-α-tubulin DM1A (diluted 1:500; Sigma-Aldrich), Alexa Fluor 488 anti-rabbit and Texas-Red anti-mouse (diluted 1:200; Invitrogen) were used. Coverslips were mounted on slide using Vectashield media containing DAPI (Vector Laboratories).

### Microscopy

Images shown in Fig. 2D were acquired on an AxioImager microscope (Carl Zeiss) with a 100x oil objective (NA 1.4 DICIII) and an AxioCam HRM camera (Carl Zeiss) using AxioVision software (Carl Zeiss).

### LUMIER assay

Half a million stably transfected Map205-RLucII/GFP10-Plk1 cells in Tyrode solution were seeded per well in a 96-well plate and allowed to settle for 30 min at 37 °C. Compounds were added to the wells (final concentration between 10–25 μM), mixed, and plates were incubated at 37 °C for 2 hours. Plates were then centrifuged at 4 °C at 3000 rpm for 5 minutes to pellet cells. Cells were then lysed with the IP buffer (20 mM Tris pH 7.5, 150 mM NaCl, 2 mM EGTA, 0.5% NP-40, 1 mM DTT, PMSF, aprotinin and leupeptin) and incubated on ice for 20 minutes. Plates were centrifuged again at 4 °C at 3400 rpm for 10 minutes, and the soluble fraction was transferred into a new 96-well plate. Note that 10 μl of this fraction was transferred in white 96-well plates from which the luminescence was read as a measure of the input amount of cell lysate for the co-IP. GFP-Plk1 was immunoprecipitated by adding 0.35 μl of anti-GFP antibody (Invitrogen). Plates were sealed and incubated on a rotator at 4 °C for 30 minutes. Then, 4 μl of pre-washed protein A magnetic beads (Invitrogen) were added to each well. Plates were sealed and incubated at 4 °C for 1h30. The extracts + beads were then transferred in a 96-well PCR plates and beads were washed 3 times with the IP buffer using a magnetic 96-well rack. The fourth wash was done in Tyrode solution, then beads were re-suspended with it and transferred in a white 96-well plate. Coelenterazine h (Biotium) was added to the beads at a final concentration of 2.5 μM. Plates were shaken and enhanced luminescence was read using an EnVision plate reader. Tyrode was also added to the plates containing the input fraction, then Coelenterazine h was added to the wells, and enhanced luminescence was read as described above. Data were analyzed as described in Table S1.

### Protein purification

Human Plk1 full length protein was purchased from Abcam and human GST-PBD_367-603_ was purchased from Sigma-Aldrich. His-PBD_326-603_ was expressed in *E.coli* BL21 using the plasmid pET28a-10His-FA-Plk1-PBD_326-603_, graciously donated by Dr. Thorsten Berg (Universität Leipzig, Germany). Cells were lysed in a buffer composed of 50 mM NaH_2_PO_4_ pH 8, 300 mM NaCl, 10 mM imidazole, 10 mM β-mercaptoethanol, PMSF, aprotinin and leupeptin. Cells were sonicated, then triton X-100 (0.5% final concentration) was added and tubes were incubated on a rotator for 20 minutes at 4 °C. PBD was purified by affinity chromatography using the Talon cobalt resin (Clontech Laboratories inc) according to the manufacturer’s procedures. Beads were washed 3 times 5 minutes with a buffer identical to the lysis buffer described above but with 600 mM NaCl and with 0.5% triton. Two supplementary washes were done using the previous buffer but with 400 mM NaCl and without triton. His-PBD was eluted with a buffer containing 50 mM NaH_2_PO_4_ pH 8, 400 mM NaCl and 1 M imidazole and adjusted to pH 8.0. His-PBD used in the FP assays was dialyzed as described^63^. His-PBD used in western blot, Coomassie Blue gel staining and mass spectrometric analyses was dialyzed three times, to decrease the presence of imidazole, against a buffer containing 50 mM NaH_2_PO_4_ pH 8, 400 mM NaCl, 1 mM EDTA, 10% glycerol and 0.01% NP-40. Note that this buffer was optimized due to the reactivity of Cpd 161 with nucleophiles (Tris, DTT, and imidazole) and was validated to have no influence on the His-PBD/phosphopeptide binding experiments assayed by fluorescence polarization (data not shown).

### Fluorescence polarization assay

The FP buffer was composed of 10 mM Tris pH 8.0, 50 mM NaCl, 1 mM EDTA, 20% glycerol and 0.1% NP-40. The peptide (final concentration 10 nM) was always added to the wells of non-binding, black, 384-well plates, 1 hour after the addition of the protein with the buffer (+/− treatment, 10% DMSO). Fluorescence polarization was then measured using a Synergy^TM^ NEO microplate reader (excitation filter: 485 nm, emission filter: 530 nm; BioTek Instruments). EC_65_ values were determined based on the binding curves, and correspond to the concentration of protein that produces 65% of increase in FP between the baseline polarization of the free peptide (absence of protein) and the completely bound state (maximal polarization). EC_65_s were used to perform the competitive assays with the non-labeled peptides and with the inhibitors. FP Inhibition was calculated with the formula: 100 − [((mP_sample_ − mP_free peptide_)\(mP_DMSO_ − mP_free peptide_)) × 100]. The fluorescent-labeled peptides used in this assay are FITC-GPMQS{T}PLNG (phosphopeptide), and FITC-GIAVPDEREFDIEADMRPHEL (Map peptide), and corresponding non-labeled peptides are identical but without FITC in N-terminal. All peptides were >95% pure and synthesized by Bio Basic inc. Inhibition curves were fitted with GraphPad (Prism software).

### Western Blotting and Coomassie Blue gel staining

His-PBD_326-603_ was incubated with different compounds for the indicated period of time at room temperature in a buffer composed of 10 mM NaH_2_PO_4_ pH 8.0, 50 mM NaCl, 1 mM EDTA, 10% glycerol and 0.01% NP-40. Laemmli 2x buffer was added to stop the reaction and samples were boiled for 5 minutes, then subjected to SDS-PAGE on 12% acrylamide gels and then blotted on nitrocellulose membranes. Membranes were blocked with TBS-Tween 0.1% containing 5% of dry milk powder, then incubated with anti-Plk1 (F-8; Santa Cruz Biotechnologies Inc.) or with a 6x-His epitope tag antibody (Invitrogen; gift from Gregory Emery). HRP-conjugated goat anti-mouse IgG (Jackson ImmunoResearch Laboratories inc.) was used and TBS-Tween 0.1% was used for the washes before and after the secondary antibody. For the Coomassie Blue gel staining, gels were first fixed for 5 minutes in a fixing solution containing 40% ethanol and 10% glacial acetic acid, stained in a solution containing 50% methanol, 10% glacial acetic acid and 0.1% Coomassie Blue, and destained with a solution of 12% ethanol and 7% glacial acetic acid. Gels were imaged using a ChemiDoc MP system (BioRad).

### Inhibitors

The compound library used for the chemical screen was provided by the IRIC HTS facility and was assembled from various commercial sources and in house syntheses (http://www.iric.ca/en/research/core-facilities/high-throughput-screening/?section=technologies). Thymoquinone was obtained from Sigma-Aldrich, Poloxin and BI2536 from MedChem Express, and PPG from ChromaDex. See Supplementary Table S2 for all vendor information concerning other compounds. All chemical structures were drawn using ChemBioDraw software.

### Chemistry

#### General methods

Unless otherwise noted, all common reagents and solvents were used as obtained from commercial suppliers without further purification. All reactions requiring anhydrous conditions were conducted under a positive pressure of nitrogen. HNMR were recorded on a 400 MHz Bruker instrument, in CDCl_3_ as solvent, with chemical shifts (δ) referenced to internal standard CDCl_3_ (7.26 ppm). LCMS were recorded on an Agilent Technologies Model 6120 quadrupole.

### Syntheses


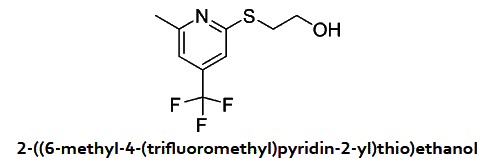


In a 20 ml sealed tube were added 2-chloro-6-methyl-4-(trifluoromethyl)pyridine (1 g, 5.11 mmol), 2-mercaptoethanol (1.800 ml, 25.6 mmol) and K_2_CO_3_ (0.989 g, 7.16 mmol) in DMF (10 ml). The mixture was heated to 80 °C for two hours. The reaction mixture was cooled down and diluted with ethyl acetate, washed with a solution of NaHCO_3_ 5%, brine and dried over Na_2_SO_4_. After evaporation of the solvents, the residue was purified on ISCO using a RediSep Gold 24 g column (Hex/EtOAc). 2-((6-methyl-4-(trifluoromethyl)pyridin-2-yl)thio) ethanol (1.20 g, 5.06 mmol, 99% yield) was obtained as an oil. ^1^H NMR (400 MHz, CDCl_3_) δ 7.32 (s, 1 H), 7.09(s, 1 H), 4.54 (brs, 1 H), 3.98 (t, *J* = 5.5 Hz, 2 H), 3.37 (t, *J* = 5.5 Hz, 2 H), 2.59 (s, 3 H). MS (APCI+) Calculated for [C_9_H_10_F_3_NOS + H]^+^: 238.1, found: 238.1.


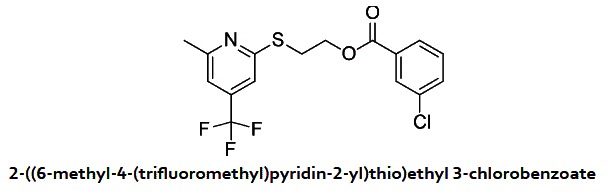


In a 10 ml round-bottomed flask were added 2-((6-methyl-4-(trifluoromethyl) pyridin-2-yl)thio)ethanol (103 mg, 0.434 mmol), pyridine (70.2 μl, 0.868 mmol), DMAP (1.061 mg, 8.68 μmol) and 3-chlorobenzoyl chloride (83 μl, 0.651 mmol) in DCM (2 ml) at 0 °C. After 2 hours, the reaction mixture was diluted with ethyl acetate, washed with a solution of NaHCO_3_ saturated, brine and dried over Na_2_SO_4_. After evaporation of the solvents, 2-((6-methyl-4-(trifluoromethyl)pyridin-2-yl)thio)ethyl 3-chlorobenzoate (155 mg, 0.412 mmol, 95% yield) was obtained as an oil. ^1^H NMR (400 MHz, CDCl_3_) δ 7.94 (m, 1 H), 7.88 (m, 1 H), 7.54 (m, 1 H), 7.37 (m, 1 H), 7.23 (s, 1 H), 7.02 (s, 1 H), 4.60 (t, *J* = 6.3 Hz, 2 H), 3.63 (t, *J* = 6.3 Hz, 2 H), 2.55 (s, 3 H). MS (APCI+) Calculated for [C_16_H_13_ClF_3_NO_2_S + H]^+^: 376.1, found: 376.1.


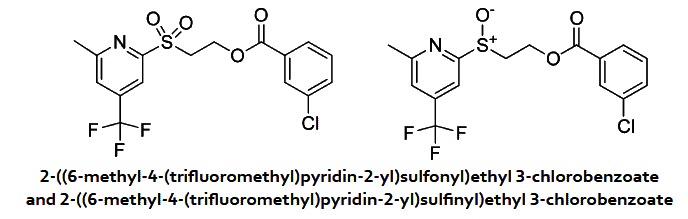


In a 10 ml round-bottomed flask were added mCPBA (40 mg, 0.178 mmol), 2-((6-methyl-4-(trifluoromethyl)pyridin-2-yl)thio)ethyl 3-chlorobenzoate (50 mg, 0.133 mmol), in DCM (2 ml). After 2 hours, the reaction mixture was diluted with ethyl acetate, washed with a solution of NaHCO_3_ saturated, brine and dried over Na_2_SO_4_. After evaporation of the solvent, the residue was purified on ISCO using a RediSep 4 g column (Hex/EtOAc). The crude product was dissolved and loaded onto a pre-column. Both products were obtained as white solids. 2-((6-methyl-4-(trifluoromethyl)pyridin-2-yl)sulfonyl)ethyl 3-chlorobenzoate (6 mg, 0.015 mmol, 11% yield) ^1^H NMR (400 MHz, CDCl_3_) δ 8.01 (s, 1 H), 7.60 (m, 1 H), 7.53 (m, 1 H), 7.46 (m, 1 H), 7.44 (s, 1 H), 7.30 (s, 1 H), 4.79 (t, *J* = 5.9 Hz, 2 H), 3.99 (t, *J* = 5.9 Hz, 2 H), 2.59 (s, 3 H) MS (APCI+) Calculated for [C_16_H_13_ClF_3_NO_4_S + H]^+^: 408.1, found: 408.1; 2-((6-methyl-4-(trifluoromethyl)pyridin-2-yl)sulfinyl)ethyl 3-chlorobenzoate (16 mg, 0.041 mmol, 31% yield) ^1^H NMR (400 MHz, CDCl_3_) δ 8.09 (s, 1 H), 7.71 (m, 1 H), 7.65 (m, 1 H), 7.53 (m, 1 H), 7.34 (m, 2 H), 4.89 (m, 1 H), 4.74 (m, 1 H), 3.59 (M, 2 H), 2.59 (s, 3 H). MS (APCI+) Calculated for [C_16_H_13_ClF_3_NO_3_S + H]^+^: 392.1, found: 392.1.


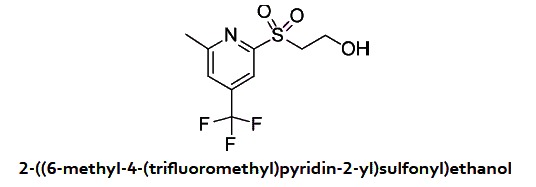


In a 10 ml round-bottomed flask was added 2-((6-methyl-4-(trifluoromethyl) pyridin-2-yl)thio)ethanol (350 mg, 1.475 mmol) in DCM (6 mL), mCPBA (827 mg, 3.69 mmol) was added in portions. After 1 hour, the reaction mixture was diluted with DCM, washed 2X with a solution of saturated sodium bicarbonate, with brine and dried over Na_2_SO_4_. After evaporation of the solvent, 2-((6-methyl-4-(trifluoromethyl)pyridin-2-yl)sulfonyl)ethanol (386 mg, 1.434 mmol, 97% yield) was obtained as an oil. ^1^H NMR (400 MHz, CDCl_3_) δ 8.17 (s, 1 H), 7.66 (s, 1 H), 4.15 (t, *J* = 5.1 Hz, 2 H), 3.67 (t, *J* = 5.1 Hz, 2 H), 3.40 (brs, 1 H), 2.77 (s, 3 H). MS (APCI+) Calculated for [C_9_H_10_F_3_NO_3_S + H]^+^: 270.1, found: 270.1.


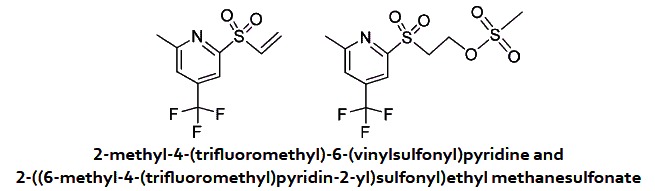


In a 10 ml round-bottomed flask were added 2-((6-methyl-4-(trifluoromethyl) pyridin-2-yl)sulfonyl)ethanol (380 mg, 1.411 mmol) and Mesyl-Cl (132 μl, 1.694 mmol) in DCM (4 mL). At 0 °C was added triethylamine (246 μl, 1.764 mmol). After 1 hour, the reaction mixture was diluted with DCM, washed with a solution of HCl 1 N, then a solution of NaHCO_3_ 5%, and with brine. The products were separated on ISCO using a RediSep 4 g column (Hex/EtOAc) and were obtained as solids. 2-methyl-4-(trifluoromethyl)-6-(vinylsulfonyl) pyridine (83 mg, 0.330 mmol, 23% yield) ^1^H NMR (400 MHz, CDCl_3_) δ 8.14 (s, 1 H), 7.60 (s, 1 H), 6.97 (dd, *J* = 9.8 and 16.8 Hz, 1 H), 6.65 (d, *J* = 16.8 Hz, 1 H), 6.28 (d, *J* = 9.8 Hz, 1 H), 2.75 (s, 3 H). MS (APCI+) Calculated for [C_9_H_8_F_3_NO_2_S + H]^+^: 252.1, found: 252.1. 2-((6-methyl-4-(trifluoromethyl) pyridin-2-yl)sulfonyl) ethyl methanesulfonate (290 mg, 0.835 mmol, 59% yield). ^1^H NMR (400 MHz, CDCl_3_) δ 8.12 (s, 1 H), 7.65 (s, 1 H), 4.68 (t, *J* = 5.8 Hz, 2 H), 3.90 (t, *J* = 5.8 Hz, 2 H), 2.99 (s, 3 H), 2.77 (s, 3 H). MS (APCI+) Calculated for [C_10_H_12_F_3_NO_5_S_2_ + H]^+^: 348.0, found: 348.0.


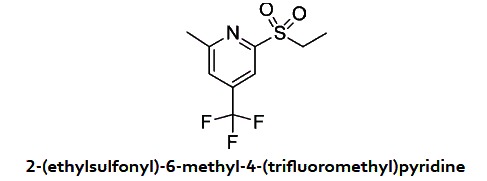


In a 5 ml vial were added 2-methyl-4-(trifluoromethyl)-6-(vinylsulfonyl)pyridine (15 mg, 0.060 mmol) and NaBH_4_ (2.259 mg, 0.060 mmol) in MeOH (0.9 mL). After 1 hour, the product was purified on prep HPLC. 2-(ethylsulfonyl)-6-methyl-4-(trifluoromethyl)pyridine (13 mg, 0.051 mmol, 86% yield) was obtained as an oil. ^1^H NMR (400 MHz, CDCl_3_) δ 8.15 (s, 1 H), 7.63 (s, 1 H), 3.48 (q, *J* = 7.4 Hz, 2 H), 2.77 (s, 3 H), 1.35 (t, *J* = 7.4 Hz, 3 H). MS (APCI+) Calculated for [C_9_H_10_F_3_NO_2_S + H]^+^: 254.1, found: 254.1.

#### Stability of Cpd 161 under slightly basic conditions

Cpd 161, which is 2-((6-methyl-4-(trifluoromethyl)pyridin-2-yl)sulfonyl)ethyl 3-chlorobenzoate (400 μM final concentration), was added to 500 μl of FP buffer (previously described). After approximately 10 minutes, 2-methyl-4-(trifluoromethyl)-6-(vinylsulfonyl) pyridine and m-Chlorobenzoic acid were observed by LC/MS.

#### Amino acids reactions with TQ, Poloxin and the vinyl sulfone derivative

To a stirred solution of electrophile (0.02 mmol) and amino acid (1- 1.5eq) in DMF (0.3 ml) were added a few drops of a diluted aqueous solution of sodium bicarbonate. The reaction was monitored using an Agilent 6120 Quadrupole LC/MS system (Agilent Technologies). In all cases, the molecular ion of the product was observed.

### Mass spectrometry

#### MALDI-TOF-MS analysis

The sinapinic acid matrix solution, used in all experiments, was prepared at saturation in a solvent mixture (water with 0.1% TFA: acetonitrile (ACN), 1:1 v/v). Acids and organic solvents used were HPLC grade or better. His-PBD_326-603_ (4 μg) was incubated with DMSO, 2.5 mM TQ or 250 μM Poloxin at room temperature in a buffer composed of 10 mM NaH_2_PO_4_ pH 8.0, 50 mM NaCl, 1 mM EDTA, 10% glycerol and 0.01% NP-40 for approximately 1–3 hours before the washing procedure, where each protein sample was concentrated and desalted by using *C4* Zip-Tips (Millipore). Protein was then eluted using a solution of ACN: water with 0.1% TFA (8:2 v/v). For the sample-matrix crystallization procedure, the aliquot of protein was then mixed with the saturated matrix in a 1:1 ratio (v/v) and 1 μl of this mixture was directly spotted onto the MALDI target plate. MALDI-TOF-MS: MALDI spectra were acquired on an UltrafleXtreme time-of-flight spectrometer operated in the positive ion, linear mode equipped with a 2 KHz repetition rate Smartbeam II laser (Bruker Daltonics). For each spectrum, 1000 shots were accumulated under optimized delayed extraction conditions with a source accelerating voltage of +20 kV, in a mass range of 10–50 kDa. BSA (Sigma-Aldrich) was used to calibrate the instrument.

#### LC-MS/MS analysis

His-PBD_326-603_ (20 μg) was incubated with DMSO, 2.5 mM TQ, 2.5 mM Cpd 161 or 250 μM Poloxin at room temperature for 3 hours in the same buffer as used in the MALDI-TOF-MS analyses. Proteins were precipitated using acetone and resuspended in 100 μl. Sample reduction was performed by adding 50 μl of 5 mM TCEP in 50 mM ammonium bicarbonate. Samples were then added 50 μl of chloroacetamide 50 mM with ammonium bicarbonate 50 mM to protect exposed cysteine residues. One microgram of trypsin was added and the digestion was performed for 8 h at 37 °C. Samples were loaded on a C_18_ stem trap (Optimize Technologies Inc) and separated on a home-made C_18_ analytical column (15 cm × 150 μm i.d.) with a 116-min gradient from 0–30% acetonitrile (0.2% FA) and a 600 nl/min flow rate on a Ultimate 3000 LC system (Dionex) connected to a Q-Exactive Plus hybrid quadrupole-orbitrap mass spectrometer (Thermo Fisher Scientific). Each full MS spectrum acquired with a 70,000 resolution was followed by 12 MS/MS spectra, where the 12 most abundant multiply charged ions were selected for MS/MS sequencing. Peptides were identified using Peaks 8.0 (Bioinformatics Solution Inc.) and peptide sequences were blasted against the Human Uniprot database. Tolerance was set at 10 ppm for precursor and 0.01 Da for fragment ions during data processing. For the post-translational modification of proteins, occurrence of carbamidomethylation (C), oxidation (M), deamidation (NQ) was considered as well as addition of TQ (C, K), Poloxin (K, C, H), and vinyl sulfone (C, H, K) when needed.

### Modeling - molecular docking

Prior to molecular docking, the Plk1 PBD coordinates, obtained from the crystal structure of the Plk1 PBD in complex with a phosphopeptide (PDB entry 1UMW^53^, chain A), have been prepared using the Structure Preparation workflow implemented in the MOE package (*Molecular Operating Environment (MOE)*, 2013.08; Chemical Computing Group Inc., 1010 Sherbooke St. West, Suite #910, Montreal, QC, Canada, H3A 2R7, 2016.) in order to model missing atomic data and add hydrogen atoms to be reflective of protonation state at physiological pH. The prepared PBD was then subjected to the AutodockTools^64^ interface to generate PDBQT docking input files and to specify the search space as a cubic volume enclosing the entire PBD structure. Three-dimensional coordinates of Cpd 161 were generated using Chemaxon Standardizer, JChem 5.1.2 (chemaxon.com). Molecular docking of Cpd 161 on Plk1 PBD was carried out using Autodock-Vina^65^. Docking poses were visually analysed using PyMOL (PyMOL Molecular Graphics System, Version 1.4 Schrödinger, LLC).

### Data analysis and structure

Raw data were analyzed using the Prism 5.00 (GraphPad) software. The structure of the zebrafish Plk1 KD and PBD in complex with a fragment of Drosophila Map205 (PDB 4J7B) was represented using PyMOL (PyMOL Molecular Graphics System, Version 1.4 Schrödinger, LLC).

## Additional Information

**How to cite this article**: Normandin, K. *et al*. Identification of Polo-like kinase 1 interaction inhibitors using a novel cell-based assay. *Sci. Rep.* **6**, 37581; doi: 10.1038/srep37581 (2016).

**Publisher's note:** Springer Nature remains neutral with regard to jurisdictional claims in published maps and institutional affiliations.

## Supplementary Material

Supplementary Information
