# Supplementary material for: Identification of Polo-like kinase 1 interaction inhibitors using a novel cell-based assay
Source: Sci Rep. 2016 Nov 22;6:37581. doi: 10.1038/srep37581 (PMC5118709; doi:10.1038/srep37581)
Supplement: Supplementary Information [file srep37581-s1.pdf]

Identification of Polo-like kinase 1 interaction inhibitors using a novel cell-based assay

Karine Normandin[1], Jean-François Lavallée[1], Marie Futter[1], Alexandre Beautrait[1], Jean Duchaine[1], Sébastien Guiral[1], Anne Marinier[1, 2] & Vincent Archambault[1, 3] *

[1]Institute for Research in Immunology and Cancer, Université de Montréal; [2]Département de chimie, Université de Montréal; [3]Département de biochimie et médecine moléculaire, Université de Montréal.

*Corresponding author: vincent.archambault.1@umontreal.ca

SUPPLEMENTARY FIGURES AND TABLES

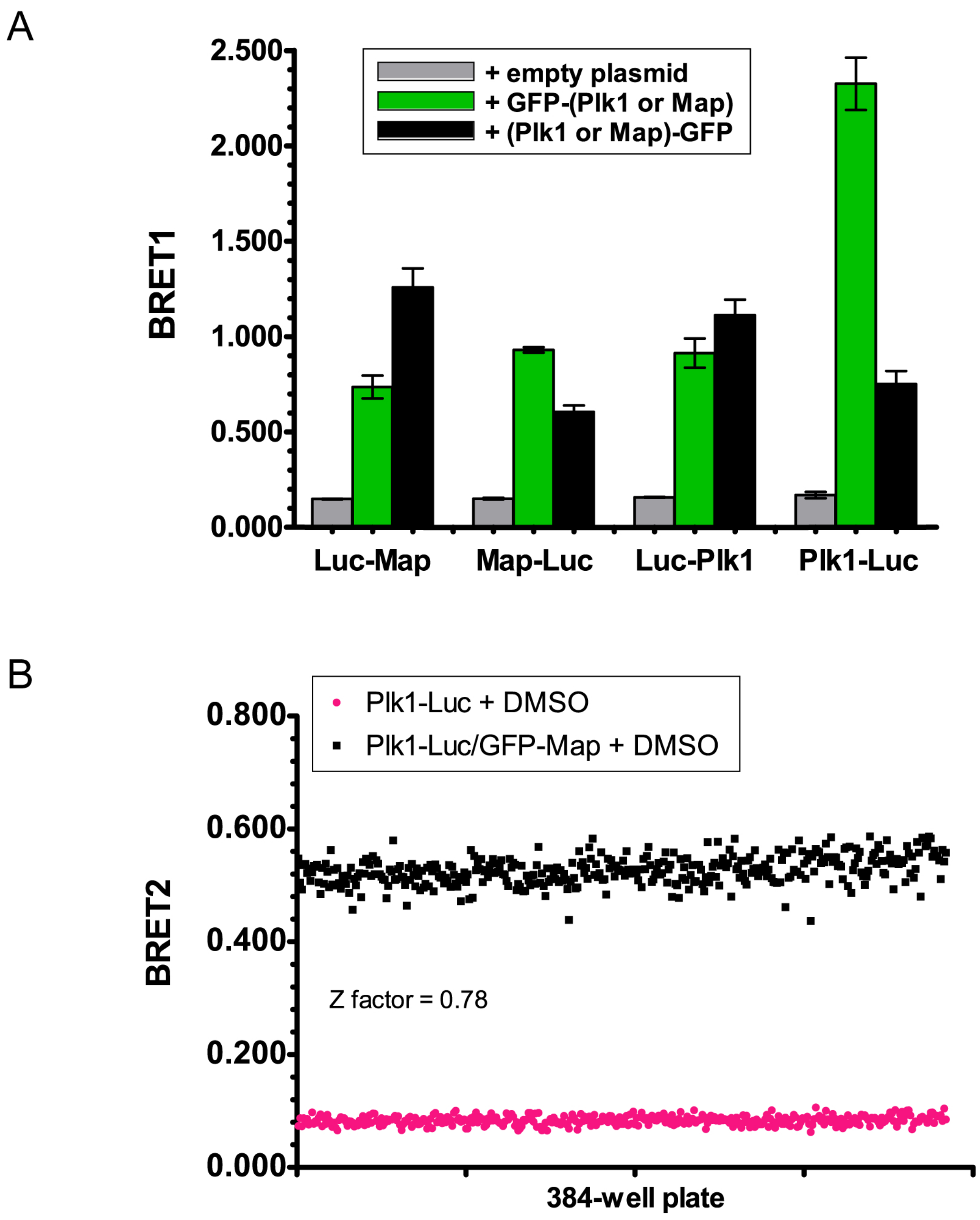

Figure S1. Development of the BRET assay used in the screen. A. BRET results for all combinations of Plk1 and $Map_{254-416}$ tagged with RLucII and GFP10 on their N-terminus or C-terminus. Experiment was performed in triplicate. Error bars: standard deviation. The Plk1-Luc/GFP-Map combination was selected because it produced the highest BRET dynamic window. Clonal stable cell lines co-expressing these proteins were then generated. B. Statistical validation of the BRET assay. One 384-well plate containing stable HEK293T Plk1-Luc/GFP-Map cells and one 384-well plate containing stable HEK293T Plk1-Luc cells (negative control) were treated for 2 hours with 0.5% DMSO (vehicle) before reading the BRET2. A Z-factor of 0.78 was obtained.

Cpd 16

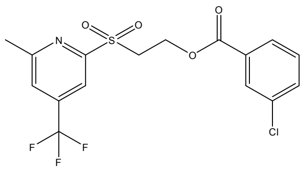

Cpd 46

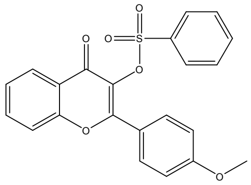

Cpd 69

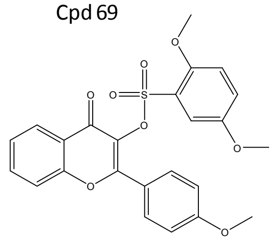

Cpd 122

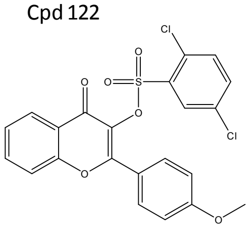

Figure S2. Chemical structures of the BRET hits also active in the mitotic index assay.

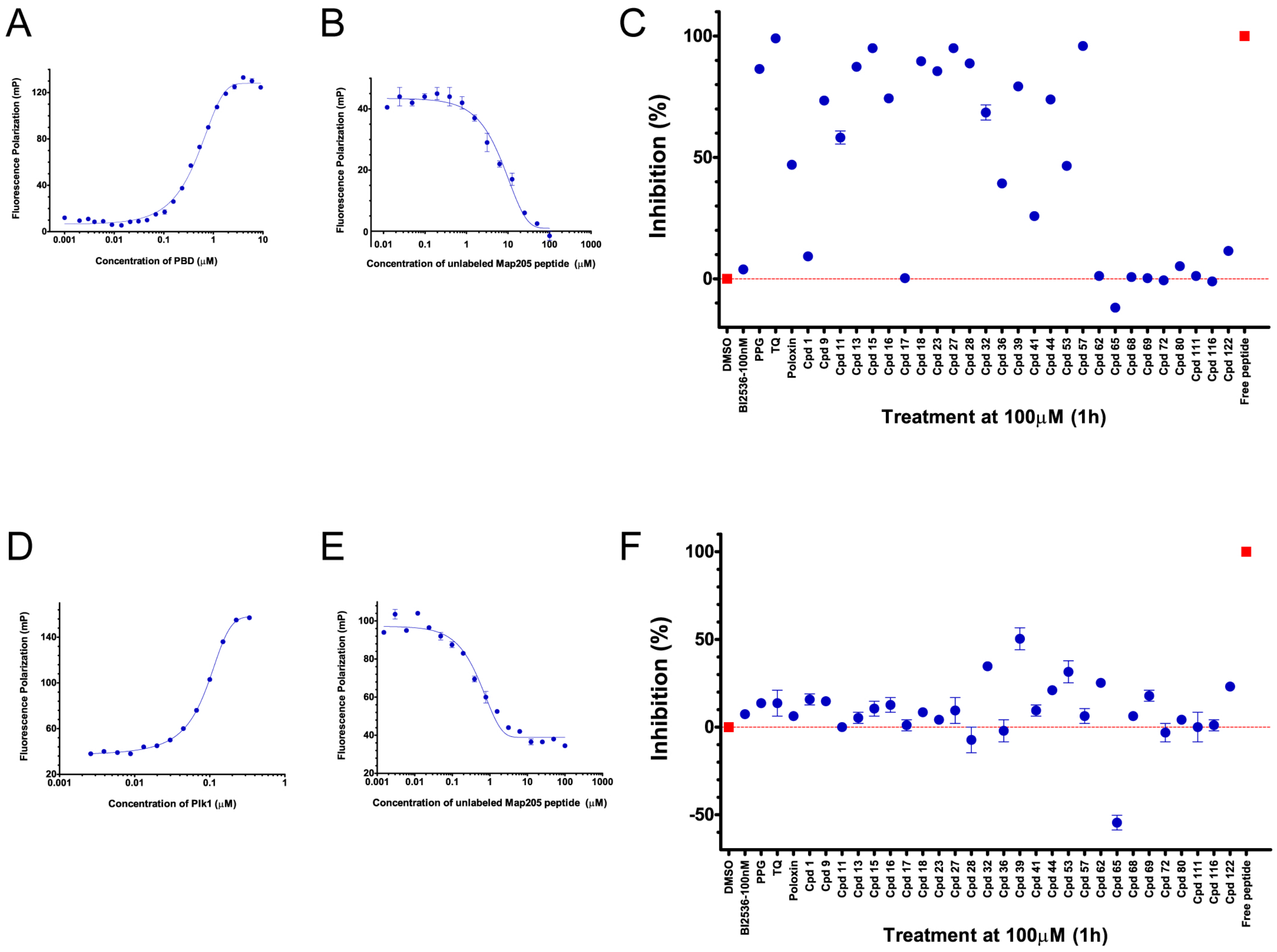

Figure S3. Compounds identified interfere with the interaction between a Map peptide and the PBD alone (A-C) or whole Plk1 (D-F) in a Fluorescence Polarization assay. A and D. Binding of the labeled FITC-Map peptide (10 nM) with increasing concentrations of His-PBD$_{326-603}$ (A) or with whole Plk1 (D). B and E. Competitive displacement of the FITC-Map peptide by the unlabeled Map peptide, added at increasing concentrations, with the Plk1 His-PBD$_{326-603}$ (used at EC$_{65}$ = 700 nM) (B) or with the whole Plk1 (used at EC$_{65}$ = 125 nM) (E). C and F. Targeted screen of selected compounds in the FP assay with the Map peptide. His-PBD$_{326-603}$ (1800 nM) (C) and whole Plk1 (130 nM) (F) were treated for 1 hour with different compounds at 100 μM before addition of the FITC-Map peptide. Values are means of duplicates from the same experiment (± range between values).

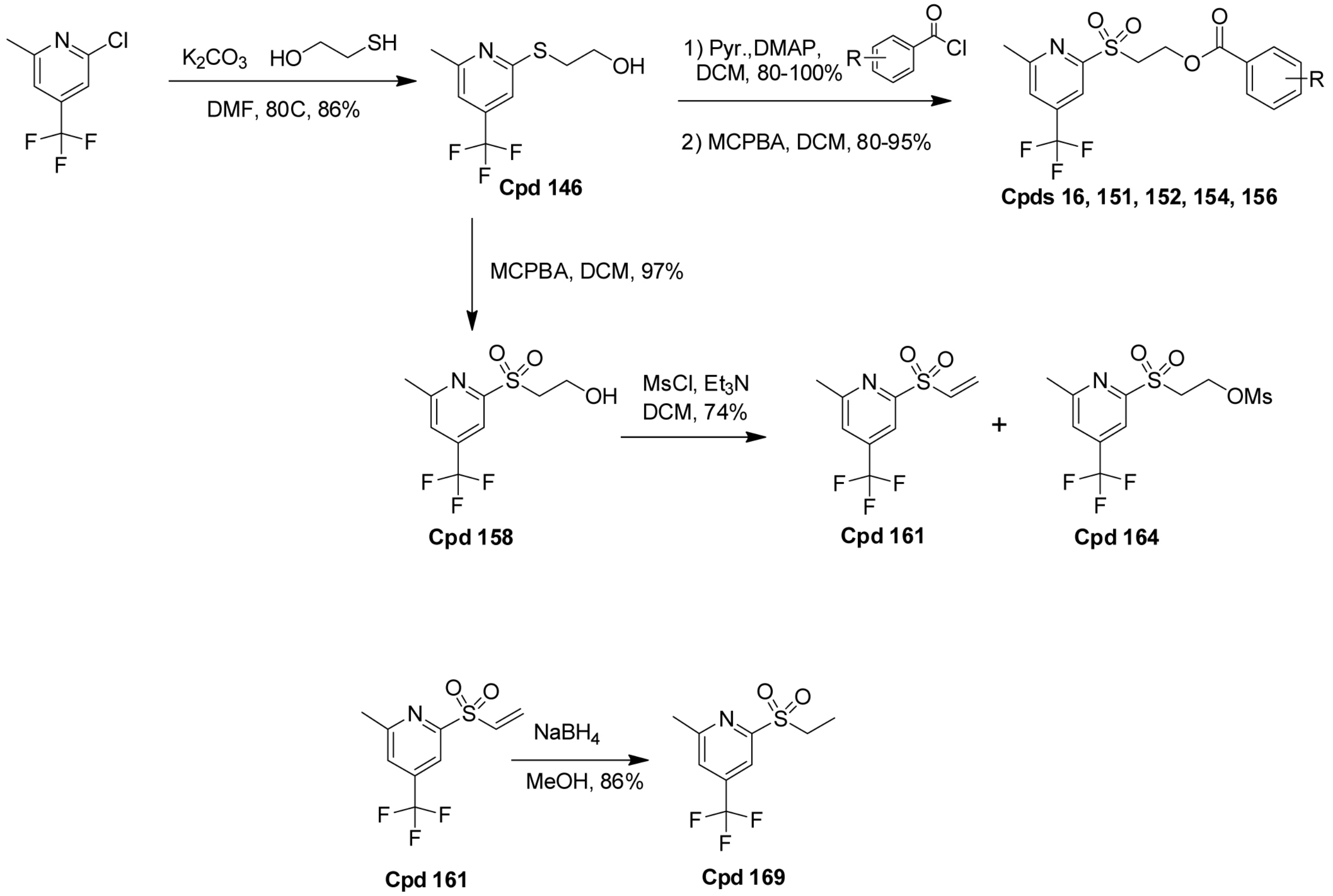

Figure S4. Synthesis schemes for SAR around Cpd 16. Other compounds were obtained commercially.

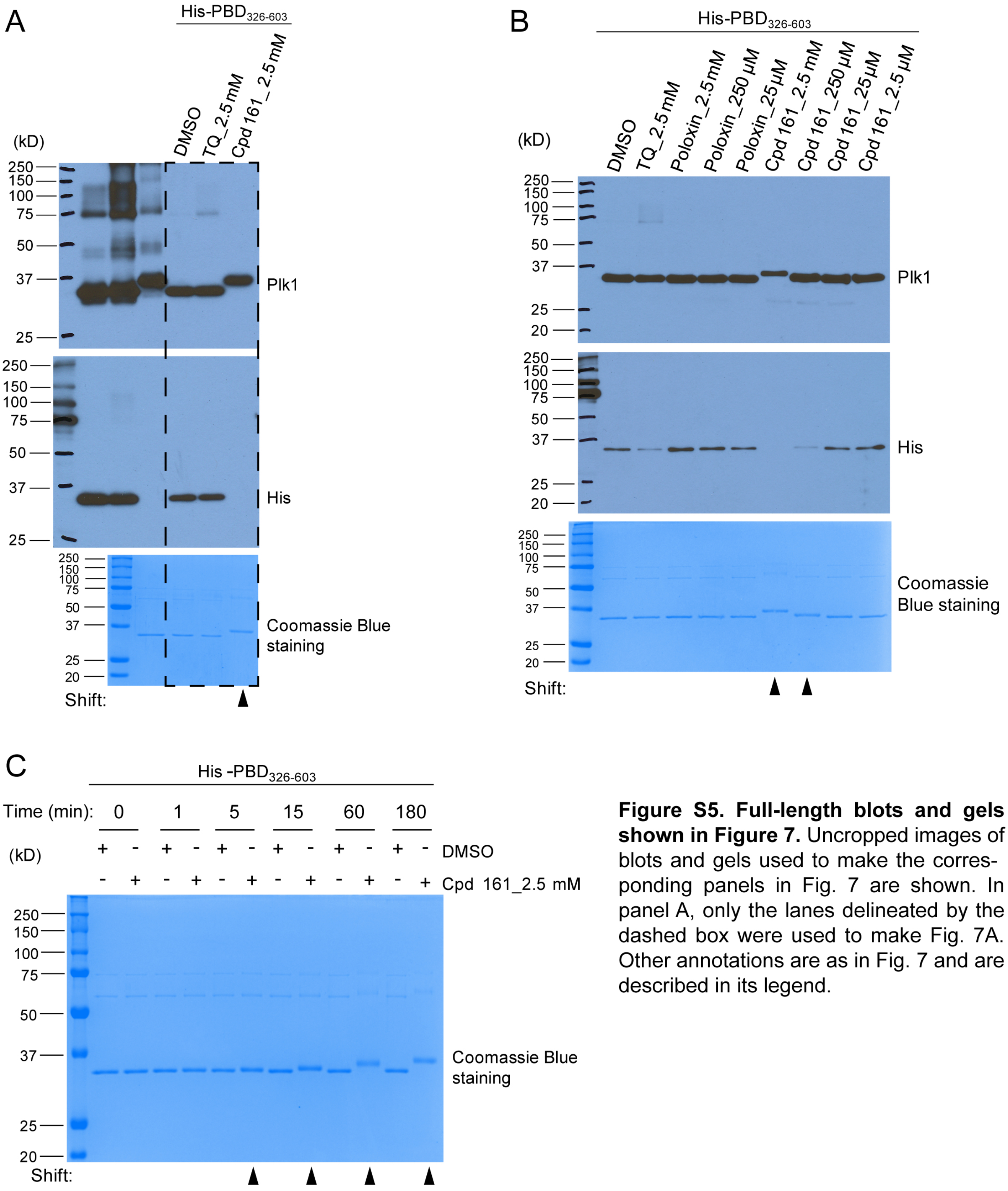

Figure S5. Full-length blots and gels shown in Figure 7. Uncropped images of blots and gels used to make the corresponding panels in Fig. 7 are shown. In panel A, only the lanes delineated by the dashed box were used to make Fig. 7A. Other annotations are as in Fig. 7 and are described in its legend.

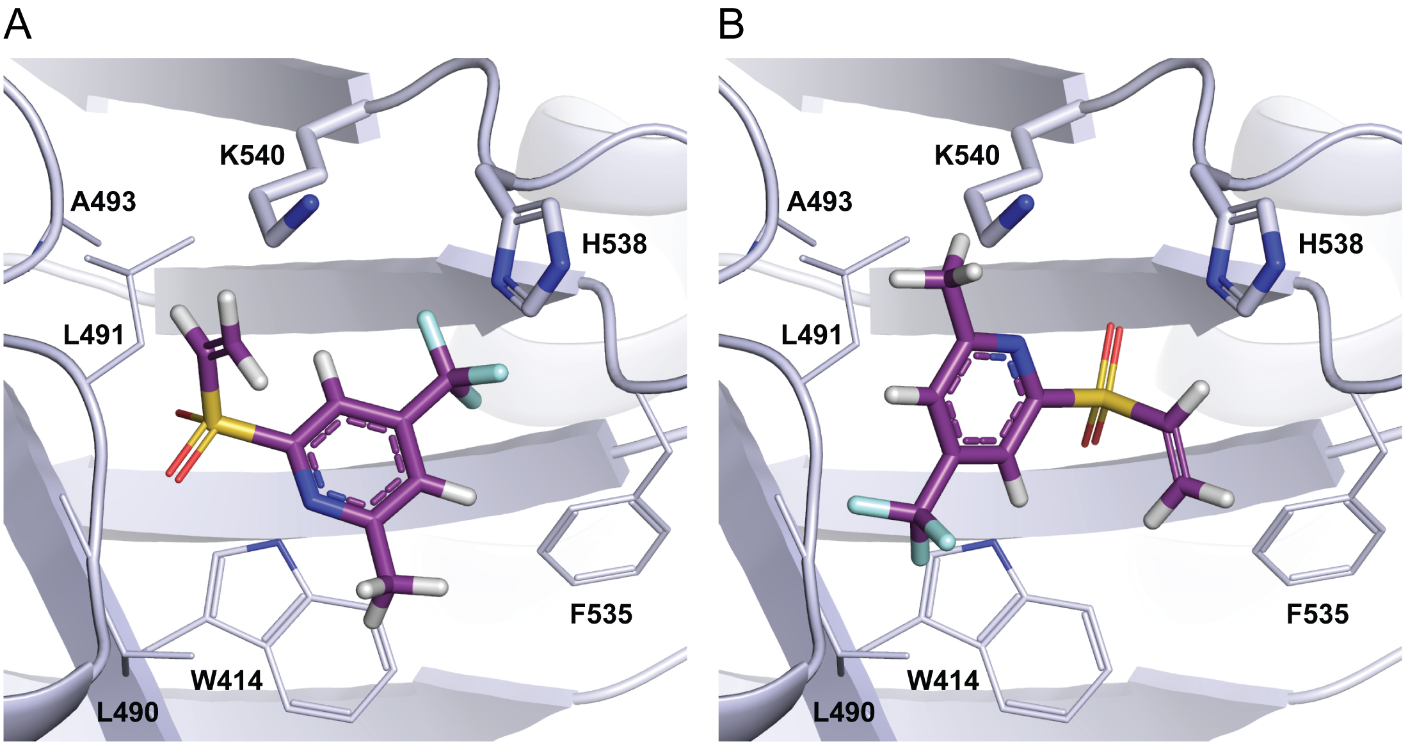

Figure S6. Putative binding modes of Cpd 161 on the PBD. Molecular docking predicts that the preferred binding site of Cpd 161 on the PBD (PDB entry 1UMW) is its phosphopeptide binding pocket (where W414, H538 and K540 are crucial residues). Two alternative poses of Cpd 161, with non-significant difference in their respective estimated docking score, are shown (A and B). PBD is shown as cartoon and sticks, carbon and nitrogen atoms are in grey and blue color, respectively. Cpd 161 is shown as sticks, colored as follows: purple: carbon atoms, blue: nitrogen, yellow: sulfur; red: oxygen, light blue: fluorine, white: hydrogen.

Table S1. Raw data from the LUMIER assay (Figure 3). Luminescence on beads are presented for each of the 4 plates (one per tab). DMSO was included as an internal control on each plate (first and last column, in gray) and all compounds were tested in duplicate (side by side) on a same plate. Due to the time needed for the readings between columns in this assay, we found that counts systematically increased from left to right in each plate, as the enzymatic reaction progressed. To circumvent this problem, we analyzed the data in the following way. The mean luminescence value for each compound was divided by the maximum mean value of each column of compounds (in orange, normalized to 1). Resulting values are expressed as Relative Luminescence Units (RLU). The data should be viewed as rough indications of activity in co-IP.

PLATE 1

BEADS

	Column #1		Column #2	Column #3		Column #4	Column #5		Column #6	Column #7		Column #8	Column #9		Column #10
**DMSO:**	390,066	**Cpd 1:**	15,076	9,654	**Cpd 9:**	74,244	87,986	**Cpd 17:**	886,164	1,022,272	**Cpd 25:**	1,428,771	1,306,371	**DMSO:**	2,585,917
**DMSO:**	453,836	**Cpd 2:**	159,976	152,582	**Cpd 10:**	1,132,468	1,141,840	**Cpd 18:**	48,113	66,908	**Cpd 26:**	1,841,391	1,130,286	**DMSO:**	2,910,820
**DMSO:**	423,655	**Cpd 3:**	669,840	695,778	**Cpd 11:**	374,254	328,854	**Cpd 19:**	18,046	28,452	**Cpd 27:**	42,504	39,770	**DMSO:**	2,967,920
**DMSO:**	359,412	**Cpd 4:**	459,296	589,011	**Cpd 12:**	641,210	771,760	**Cpd 20:**	1,032,000	1,384,114	**Cpd 28:**	470,276	374,830	**DMSO:**	2,979,922
**DMSO:**	401,916	**Cpd 5:**	723,890	358,615	**Cpd 13:**	38,822	51,866	**Cpd 21:**	728,406	1,095,957	**Cpd 29:**	2,579,044	2,456,177	**DMSO:**	2,864,320
**DMSO:**	425,204	**Cpd 6:**	221,644	208,600	**Cpd 14:**	1,055,362	1,184,868	**Cpd 22:**	1,037,198	1,434,797	**Cpd 30:**	1,964,228	1,946,733	**DMSO:**	2,482,486
**DMSO:**	398,126	**Cpd 7:**	422,540	537,002	**Cpd 15:**	18,060	19,824	**Cpd 23:**	75,154	249,262	**Cpd 31:**	1,563,040	639,044	**DMSO:**	2,315,537
	57,336	**Cpd 8:**	114,916	163,133	**Cpd 16:**	21,904	28,648	**Cpd 24:**	315,073	464,828	**Cpd 32:**	26,500	26,938		217,502

	Average-Beads
**DMSO- Column #1**	407,459
**DMSO- Column #10**	2,729,560

		Relative Luminescence Unit
**DMSO**		0.907
**Cpd 1**	12,365	0.018
**Cpd 2**	156,279	0.229
**Cpd 3**	682,809	1.000
**Cpd 4**	524,154	0.768
**Cpd 5**	541,253	0.793
**Cpd 6**	215,122	0.315
**Cpd 7**	479,771	0.703
**Cpd 8**	139,025	0.204
**Cpd 9**	81,115	0.071
**Cpd 10**	1,137,154	1.000
**Cpd 11**	351,554	0.309
**Cpd 12**	706,485	0.621
**Cpd 13**	45,344	0.040
**Cpd 14**	1,120,115	0.985
**Cpd 15**	18,942	0.017
**Cpd 16**	25,276	0.022
**Cpd 17**	954,218	0.772
**Cpd 18**	57,511	0.047
**Cpd 19**	23,249	0.019
**Cpd 20**	1,208,057	0.977
**Cpd 21**	912,182	0.738
**Cpd 22**	1,235,998	1.000
**Cpd 23**	162,208	0.131
**Cpd 24**	389,951	0.315
**Cpd 25**	1,367,571	0.543
**Cpd 26**	1,485,839	0.590
**Cpd 27**	41,137	0.016
**Cpd 28**	422,553	0.168
**Cpd 29**	2,517,611	1.000
**Cpd 30**	1,955,481	0.777
**Cpd 31**	1,101,042	0.437
**Cpd 32**	26,719	0.011

**DMSO**	Column #1	Column #10	**Average**
	0.859	0.868	
	1.000	0.977	
	0.933	0.996	
	0.792	1.000	
	0.886	0.961	
	0.937	0.833	
	0.877	0.777	0.907

PLATE 2

BEADS

Column #1			Column #2	Column #3		Column #4	Column #5		Column #6	Column #7		Column #8	Column #9		Column #10
**DMSO:**	467,370	**Cpd 33:**	681,071	421,712	**Cpd 41:**	44,182	81,310	**Cpd 49:**	1,167,230	1,294,606	**Cpd 57:**	40,968	73,000	**DMSO:**	1,701,762
**DMSO:**	582,677	**Cpd 34:**	428,644	568,092	**Cpd 42:**	863,290	1,410,060	**Cpd 50:**	1,045,308	927,518	**Cpd 58:**	3,263,326	3,402,838	**DMSO:**	1,696,280
**DMSO:**	521,296	**Cpd 35:**	979,956	1,207,571	**Cpd 43:**	1,101,437	1,094,104	**Cpd 51:**	939,792	902,486	**Cpd 59:**	2,047,466	2,427,149	**DMSO:**	2,104,498
**DMSO:**	568,680	**Cpd 36:**	76,196	88,128	**Cpd 44:**	804,820	998,832	**Cpd 52:**	837,456	1,133,424	**Cpd 60:**	1,817,867	2,478,250	**DMSO:**	2,287,766
**DMSO:**	553,748	**Cpd 37:**	665,926	1,047,022	**Cpd 45:**	1,340,842	1,309,842	**Cpd 53:**	556,422	586,142	**Cpd 61:**	1,789,324	2,256,664	**DMSO:**	2,730,653
**DMSO:**	467,094	**Cpd 38:**	722,513	959,044	**Cpd 46:**	584,340	619,608	**Cpd 54:**	746,228	1,283,113	**Cpd 62:**	1,444,410	2,073,600	**DMSO:**	2,541,782
**DMSO:**	535,678	**Cpd 39:**	66,264	109,924	**Cpd 47:**	463,475	812,822	**Cpd 55:**	587,900	1,095,266	**Cpd 63:**	1,340,537	2,218,073	**DMSO:**	2,516,589
	258,198	**Cpd 40:**	227,542	285,071	**Cpd 48:**	2,760,910	2,724,993	**Cpd 56:**	608,977	913,864	**Cpd 64:**	858,840	1,249,824		990,406

	Average-Beads
**DMSO- Column #1**	528,078
**DMSO- Column #10**	2,225,619

		Relative Luminescence Unit
**DMSO**		0.861
**Cpd 33**	551,392	0.504
**Cpd 34**	498,368	0.456
**Cpd 35**	1,093,764	1.000
**Cpd 36**	82,162	0.075
**Cpd 37**	856,474	0.783
**Cpd 38**	840,779	0.769
**Cpd 39**	88,094	0.081
**Cpd 40**	256,307	0.234
**Cpd 41**	62,746	0.023
**Cpd 42**	1,136,675	0.414
**Cpd 43**	1,097,771	0.400
**Cpd 44**	901,826	0.329
**Cpd 45**	1,325,342	0.483
**Cpd 46**	601,974	0.219
**Cpd 47**	638,149	0.233
**Cpd 48**	2,742,952	1.000
**Cpd 49**	1,230,918	1.000
**Cpd 50**	986,413	0.801
**Cpd 51**	921,139	0.748
**Cpd 52**	985,440	0.801
**Cpd 53**	571,282	0.464
**Cpd 54**	1,014,671	0.824
**Cpd 55**	841,583	0.684
**Cpd 56**	761,421	0.619
**Cpd 57**	56,984	0.017
**Cpd 58**	3,333,082	1.000
**Cpd 59**	2,237,308	0.671
**Cpd 60**	2,148,059	0.644
**Cpd 61**	2,022,994	0.607
**Cpd 62**	1,759,005	0.528
**Cpd 63**	1,779,305	0.534
**Cpd 64**	1,054,332	0.316

**DMSO**	Column #1	Column #10	**Average**
	0.802	0.623	
	1.000	0.621	
	0.895	0.771	
	0.976	0.838	
	0.950	1.000	
	0.802	0.931	
	0.919	0.922	0.861

PLATE 3

BEADS

	Column #1		Column #2	Column #3		Column #4	Column #5		Column #6	Column #7		Column #8	Column #9		Column #10	Column #11
**DMSO:**	149,068	**Cpd 65:**	4,502	6,462	**Cpd 73:**	449,800	511,712	**Cpd 81:**	227,038	247,708	**Cpd 89:**	325,044	362,996	**Cpd 97:**	528,542	681,268
**DMSO:**	140,140	**Cpd 66:**	175,566	226,524	**Cpd 74:**	405,492	355,371	**Cpd 82:**	311,754	318,770	**Cpd 90:**	536,006	498,564	**Cpd 98:**	492,874	816,915
**DMSO:**	129,470	**Cpd 67:**	290,576	360,997	**Cpd 75:**	424,156	532,396	**Cpd 83:**	424,392	454,828	**Cpd 91:**	593,357	451,615	**Cpd 99:**	410,970	289,453
**DMSO:**	130,420	**Cpd 68:**	109,900	138,380	**Cpd 76:**	444,633	594,914	**Cpd 84:**	356,900	434,891	**Cpd 92:**	600,246	595,235	**Cpd 100:**	689,748	497,264
**DMSO:**	116,602	**Cpd 69:**	232,744	331,226	**Cpd 77:**	477,280	570,393	**Cpd 85:**	454,990	421,466	**Cpd 93:**	565,666	439,402	**DMSO:**	629,410	609,232
**DMSO:**	128,070	**Cpd 70:**	265,246	317,708	**Cpd 78:**	287,174	461,071	**Cpd 86:**	299,360	479,828	**Cpd 94:**	521,318	506,278	**DMSO:**	613,608	387,714
**DMSO:**	115,826	**Cpd 71:**	165,275	238,710	**Cpd 79:**	295,998	343,468	**Cpd 87:**	266,608	435,000	**Cpd 95:**	407,448	414,768	**DMSO:**	413,108	334,016
	12,484	**Cpd 72:**	64,794	178,690	**Cpd 80:**	121,111	185,792	**Cpd 88:**	170,771	302,793	**Cpd 96:**	146,777	357,380	**DMSO:**	321,470	67,770

	Average-Beads
**DMSO- Column #1**	129,942
**DMSO- Columns #10-11**	472,651

**DMSO**	Column #1	Columns #10-11	**Average**
	1.000	1.000	
	0.940	0.975	
	0.869	0.656	
	0.875	0.511	
	0.782	1.000	
	0.859	0.636	
	0.777	0.548	0.816

		**Relative Luminescence Unit**
**DMSO**		0.816
**Cpd 65**	5,482	0.017
**Cpd 66**	201,045	0.617
**Cpd 67**	325,787	1.000
**Cpd 68**	124,140	0.381
**Cpd 69**	281,985	0.866
**Cpd 70**	291,477	0.895
**Cpd 71**	201,993	0.620
**Cpd 72**	121,742	0.374
**Cpd 73**	480,756	0.918
**Cpd 74**	380,432	0.726
**Cpd 75**	478,276	0.913
**Cpd 76**	519,774	0.992
**Cpd 77**	523,837	1.000
**Cpd 78**	374,123	0.714
**Cpd 79**	319,733	0.610
**Cpd 80**	153,452	0.293
**Cpd 81**	237,373	0.540
**Cpd 82**	315,262	0.717
**Cpd 83**	439,610	1.000
**Cpd 84**	395,896	0.901
**Cpd 85**	438,228	0.997
**Cpd 86**	389,594	0.886
**Cpd 87**	350,804	0.798
**Cpd 88**	236,782	0.539
**Cpd 89**	344,020	0.576
**Cpd 90**	517,285	0.865
**Cpd 91**	522,486	0.874
**Cpd 92**	597,741	1.000
**Cpd 93**	502,534	0.841
**Cpd 94**	513,798	0.860
**Cpd 95**	411,108	0.688
**Cpd 96**	252,079	0.422
**Cpd 97**	604,905	0.924
**Cpd 98**	654,895	1.000
**Cpd 99**	350,212	0.535
**Cpd 100**	593,506	0.906

PLATE 4

BEADS

	Column #1		Column #2	Column #3		Column #4	Column #5		Column #6	Column #7		Column #8	Column #9		Column #12
**DMSO:**	250,062	**Cpd 101:**	377,313	353,900	**Cpd 109:**	275,224	381,837	**Cpd 117:**	370,854	531,853	**TQ:**	221,826	304,556	**DMSO:**	1,595,057
**DMSO:**	259,951	**Cpd 102:**	412,144	595,808	**Cpd 110:**	555,674	535,226	**Cpd 118:**	757,471	832,352	**Poloxin:**	1,325,160	1,298,916	**DMSO:**	1,442,344
**DMSO:**	228,044	**Cpd 103:**	400,971	520,906	**Cpd 111:**	96,962	94,360	**Cpd 119:**	801,555	773,018				**DMSO:**	1,414,942
**DMSO:**	286,524	**Cpd 104:**	425,618	630,168	**Cpd 112:**	702,052	676,557	**Cpd 120:**	888,117	828,624				**DMSO:**	1,095,800
**DMSO:**	185,230	**Cpd 105:**	415,974	547,728	**Cpd 113:**	690,682	745,816	**Cpd 121:**	783,868	856,250	**Cpd 122:**	909,984	1,127,164	**DMSO:**	1,122,604
**DMSO:**	207,044	**Cpd 106:**	316,228	486,338	**Cpd 114:**	557,726	625,664							**DMSO:**	1,067,646
**DMSO:**	216,178	**Cpd 107:**	246,040	392,276	**Cpd 115:**	364,440	560,460	**BI 2536:**	47,611	56,678				**DMSO:**	877,613
	107,904	**Cpd 108:**	175,324	197,258	**Cpd 116:**	248,798	364,698	**PPG:**	290,044	312,040					359,786

	Average-Beads
**DMSO- Column #1**	233,290
**DMSO- Column #12**	1,230,858

		Relative Luminescence Unit
**DMSO**		0.793
**Cpd 101**	365,607	0.693
**Cpd 102**	503,976	0.955
**Cpd 103**	460,939	0.873
**Cpd 104**	527,893	1.000
**Cpd 105**	481,851	0.913
**Cpd 106**	401,283	0.760
**Cpd 107**	319,158	0.605
**Cpd 108**	186,291	0.353
**Cpd 109**	328,531	0.457
**Cpd 110**	545,450	0.759
**Cpd 111**	95,661	0.133
**Cpd 112**	689,305	0.960
**Cpd 113**	718,249	1.000
**Cpd 114**	591,695	0.824
**Cpd 115**	462,450	0.644
**Cpd 116**	306,748	0.427
**Cpd 117**	451,354	0.526
**Cpd 118**	794,912	0.926
**Cpd 119**	787,287	0.917
**Cpd 120**	858,371	1.000
**Cpd 121**	820,059	0.955
**BI 2536**	52,145	0.061
**PPG**	301,042	0.351
**TQ**	263,191	0.201
**Poloxin**	1,312,038	1.000
**Cpd 122**	1,018,574	0.776

**DMSO**	Column #1	Column #12	**Average**
	0.873	1.000	
	0.907	0.904	
	0.796	0.887	
	1.000	0.687	
	0.646	0.704	
	0.723	0.669	
	0.754	0.550	0.793

Table S2. BRET, mitotic index and co-IP results compilation for the 122 BRET hits.

					Primary screen Plk1-Map (stable)		Primary screen CRAF-BRAF (transient)		Confirmation Plk1-Map (stable)		Confirmation Plk1-Map (transient)		Confirmation CRAF-BRAF (transient)		Mitotic index		Co-IP
Cpd #	ID	Batch	Supplier	Supplier ID	% Inihibition	% Quench RLuc	% Inihibition	% Quench RLuc	% Inihibition	% Quench RLuc	% Inihibition	% Quench RLuc	% Inihibition	% Quench RLuc	Mitotic index (%)	Nb of analyzed cells	Values relative to top in plate column = 1
1	UM0107177	01	MAYBRIDGE	AW 00482	50.82	79.13	21.44	80.08	66.71	81.02	63.61	69.40	-24.74	72.14	1.59	668	0.018
2	UM0107221	01	MAYBRIDGE	AW 00592	55.07	85.96	31.45	75.12	35.46	90.44	-34.13	73.50	0.35	69.42	5.64	1121	0.229
3	UM0107266	01	MAYBRIDGE	AW 01093	53.30	85.61	61.48	88.22	30.16	93.58	-12.13	89.74	2.18	86.31	5.16	1241	1.000
4	UM0107457	01	MAYBRIDGE	BTB 01468	61.37	74.20	6.39	64.29	37.02	71.62	3.89	51.50	-23.24	41.54	3.78	1030	0.768
5	UM0107465	01	MAYBRIDGE	BTB 01397	65.21	59.62	23.57	38.70	45.45	61.07	17.66	-13.30	-0.79	23.13	0.41	638	0.793
6	UM0107466	01	MAYBRIDGE	BTB 01470	62.68	87.76	7.51	84.96	32.04	89.40	5.59	80.38	-33.37	75.46	2.84	1005	0.315
7	UM0107535	01	MAYBRIDGE	BTB 02075	58.30	64.35	-7.01	66.17	26.54	47.47	-9.57	16.31	-35.15	6.23	4.37	811	0.703
8	UM0108212	01	MAYBRIDGE	BTB 04098	54.13	66.53	13.33	68.83	25.74	60.04	7.94	28.16	-18.98	25.97	2.89	1083	0.204
9	UM0108871	01	MAYBRIDGE	BTB 04426	56.34	77.93	-52.46	69.98	49.28	70.98	60.77	58.61	9.16	81.03	0.17	592	0.071
10	UM0024677	03	MAYBRIDGE	BTB 04957	67.60	93.78	36.31	85.60	42.67	93.83	-14.46	73.44	-11.38	63.73	4.31	1024	1.000
11	UM0111426	01	MAYBRIDGE	BTB 06019	46.60	76.68	1.34	48.60	29.73	77.70	-13.92	40.97	-10.42	53.70	7.54	910	0.309
12	UM0112103	01	MAYBRIDGE	BTB 06776	68.25	89.25	38.71	86.66	20.39	81.39	7.11	70.27	-12.51	63.67	4.08	1178	0.621
13	UM0112687	01	MAYBRIDGE	BTB 07155	76.83	85.35	51.15	84.05	61.45	79.50	68.19	66.43	18.66	83.95	3.70	612	0.040
14	UM0113547	01	MAYBRIDGE	BTB 08799	67.58	86.85	39.91	80.51	41.26	89.74	-38.11	59.00	-14.66	39.49	5.24	1125	0.985
15	UM0113559	01	MAYBRIDGE	BTB 08960	67.91	84.93	31.50	81.13	41.55	78.03	53.01	72.97	-4.59	86.14	0.81	823	0.017
16	UM0113574	01	MAYBRIDGE	BTB 09085	67.34	86.24	52.83	88.39	30.79	81.37	30.06	74.57	0.37	80.17	15.73	789	0.022
17	UM0114221	01	MAYBRIDGE	CD 03862	64.09	91.79	17.43	85.48	62.50	91.30	3.77	83.77	-44.82	79.36	9.70	843	0.772
18	UM0114440	01	MAYBRIDGE	CD 08589	45.55	78.56	8.26	87.41	42.59	81.79	60.17	69.60	7.13	87.60	0.54	458	0.047
19	UM0044124	02	MAYBRIDGE	DSHS 00690	77.53	88.69	42.12	87.63	36.81	92.52	10.92	83.45	-26.61	81.39	0.57	585	0.019
20	UM0114956	01	MAYBRIDGE	DSHS 00902	46.34	87.87	54.93	89.27	21.53	91.98	-17.19	86.96	-22.73	82.72	5.77	1281	0.977
21	UM0114962	01	MAYBRIDGE	DSHS 00691	45.76	78.45	23.02	53.92	23.26	69.35	-13.00	46.29	-15.44	29.17	5.77	1201	0.738
22	UM0115060	01	MAYBRIDGE	GK 00932	57.37	84.54	33.24	85.72	41.81	95.30	-1.29	89.97	-2.53	87.37	4.14	1226	1.000
23	UM0044370	02	MAYBRIDGE	GK 01179	78.41	82.19	-37.15	81.61	48.10	81.45	64.99	70.40	-65.02	83.59	2.63	797	0.131
24	UM0115252	01	MAYBRIDGE	GK 02719	68.10	88.13	54.62	87.02	36.96	93.36	5.68	86.41	5.36	88.72	5.59	1240	0.315
25	UM0115281	01	MAYBRIDGE	GK 03147	45.23	89.37	53.14	89.82	26.07	94.57	-8.10	92.30	-8.57	88.81	4.78	1116	0.543
26	UM0115294	01	MAYBRIDGE	GK 03068	52.76	87.32	36.28	78.36	40.41	87.86	-6.40	79.33	-8.86	73.75	3.92	1083	0.590
27	UM0115297	01	MAYBRIDGE	GK 03183	80.81	80.09	31.44	87.95	39.21	79.07	68.50	70.64	18.98	87.05	2.84	705	0.016
28	UM0044649	02	MAYBRIDGE	HAN 00116	45.62	78.12	9.04	88.52	50.48	80.87	64.88	70.41	9.73	87.46	2.87	764	0.168
29	UM0115430	01	MAYBRIDGE	HAN 00410	35.64	79.81	4.37	39.68	27.83	77.47	-13.59	29.30	-17.95	16.64	3.94	1057	1.000
30	UM0115588	01	MAYBRIDGE	HTS 01167	50.70	82.37	26.25	82.76	38.26	90.34	0.67	84.37	-16.55	76.63	4.46	1130	0.777
31	UM0045020	02	MAYBRIDGE	HTS 01731	59.16	85.22	35.04	72.15	53.24	87.56	-1.36	67.10	8.69	55.48	5.27	1336	0.437
32	UM0115695	01	MAYBRIDGE	HTS 01716	82.98	78.54	52.78	85.20	32.39	61.04	10.87	55.86	-15.05	34.17	6.85	1221	0.011
33	UM0045568	02	MAYBRIDGE	HTS 04586	46.30	21.41	9.33	66.80	25.44	52.76	3.17	40.96	-7.50	25.32	5.01	895	0.504
34	UM0116166	01	MAYBRIDGE	HTS 05440	47.36	37.79	35.79	59.50	32.78	64.09	9.76	34.37	4.03	13.92	5.87	1113	0.456
35	UM0116479	01	MAYBRIDGE	HTS 07461	47.28	41.69	13.69	17.06	33.04	51.36	-2.19	-5.46	4.61	-12.42	6.35	1023	1.000
36	UM0116537	01	MAYBRIDGE	HTS 07564	51.44	43.67	14.13	58.96	38.28	47.30	52.07	8.81	6.72	33.27	0.04	812	0.075
37	UM0046711	02	MAYBRIDGE	HTS 11170	45.24	80.82	30.49	82.80	26.10	87.33	-14.06	77.84	-17.55	62.37	5.51	1278	0.783
38	UM0117516	01	MAYBRIDGE	JFD 02414	51.01	63.14	12.78	19.29	43.35	69.42	0.03	6.66	3.22	2.00	4.51	1105	0.769
39	UM0117931	01	MAYBRIDGE	KM 01532	46.92	58.25	38.21	68.10	28.92	72.06	39.04	13.68	-28.02	51.27	1.85	915	0.081
40	UM0107759	01	MAYBRIDGE	KM 03640	57.91	59.70	3.04	65.18	33.00	64.89	28.30	40.55	15.17	70.46	0.76	711	0.234
41	UM0107915	01	MAYBRIDGE	KM 05478	48.25	76.91	9.15	82.74	22.85	91.45	-1.55	83.71	-0.82	89.16	0.65	752	0.023
42	UM0107958	01	MAYBRIDGE	KM 05637	46.57	80.72	33.05	84.16	25.51	75.79	-14.59	37.29	-5.11	13.72	4.64	1146	0.414
43	UM0109122	01	MAYBRIDGE	NRB 04070	54.41	82.81	6.29	68.33	29.95	84.93	35.79	79.11	10.00	84.92	0.38	961	0.400
44	UM0050240	02	MAYBRIDGE	NRB 04635	46.80	65.81	27.75	81.91	31.30	73.89	46.34	65.89	4.73	75.24	6.66	844	0.329

					Primary screen Plk1-Map (stable)		Primary screen CRAF-BRAF (transient)		Confirmation Plk1-Map (stable)		Confirmation Plk1-Map (transient)		Confirmation CRAF-BRAF (transient)		Mitotic index		Co-IP
Cpd #	ID	Batch	Supplier	Supplier ID	% Inhibition	% Quench RLuc	% Inhibition	% Quench RLuc	% Inhibition	% Quench RLuc	% Inhibition	% Quench RLuc	% Inhibition	% Quench RLuc	Mitotic index (%)	Nb of analyzed cells	Values relative to top in plate column = 1
45	UM0110305	01	MAYBRIDGE	RH 01618	45.20	71.08	1.10	51.49	33.67	85.08	-30.22	54.24	-9.24	31.93	5.38	1008	0.483
46	UM0110531	01	MAYBRIDGE	RJC 01628	53.18	82.37	16.40	63.54	30.35	91.04	-29.25	72.57	19.45	66.12	29.44	831	0.219
47	UM0111224	01	MAYBRIDGE	S 04076	45.29	83.95	5.83	80.16	25.06	89.54	-34.97	71.30	7.01	59.13	5.81	1150	0.233
48	UM0111382	01	MAYBRIDGE	S 09528	64.65	53.86	-17.19	85.32	33.05	76.00	66.76	64.63	-36.81	71.69	2.86	844	1.000
49	UM0111558	01	MAYBRIDGE	S 12721	47.04	83.98	26.90	82.37	22.76	94.87	-25.39	85.65	-4.13	80.84	5.42	1246	1.000
50	UM0053923	02	MAYBRIDGE	SEW 02094	50.27	81.17	30.34	74.95	21.62	89.00	-26.33	68.27	4.23	62.33	4.49	1043	0.801
51	UM0112278	01	MAYBRIDGE	SEW 04487	45.29	68.33	38.92	79.48	23.19	82.72	-21.49	67.09	-13.15	65.32	5.08	1140	0.748
52	UM0054719	02	MAYBRIDGE	SEW 06560	51.80	71.42	33.34	75.79	34.00	87.94	-7.60	68.31	1.45	68.36	5.54	1251	0.801
53	UM0054792	02	MAYBRIDGE	SP 00278	73.92	65.68	-13.36	70.12	60.53	70.21	60.20	19.84	-23.26	57.92	0.25	711	0.464
54	UM0112835	01	MAYBRIDGE	SP 01000	53.70	67.85	37.49	80.97	41.04	90.19	-32.83	72.33	-10.41	72.90	4.26	1083	0.824
55	UM0112874	01	MAYBRIDGE	SP 00932	65.38	72.24	36.23	82.49	28.65	80.09	-12.63	42.73	11.99	57.62	5.27	1126	0.684
56	UM0112902	01	MAYBRIDGE	SP 01131	67.10	77.26	28.17	81.60	50.83	90.14	-16.36	57.34	6.88	51.70	4.98	1253	0.619
57	UM0113055	01	MAYBRIDGE	SPB 01974	53.19	68.79	4.09	89.46	50.51	72.14	71.14	50.57	-10.70	69.51	0.54	716	0.017
58	UM0092330	01	SPECS	AE-842/33004023	65.44	83.13	31.26	89.59	53.82	91.81	30.67	78.98	-23.16	86.72	3.75	918	1.000
59	UM0098743	01	SPECS	AH-487/14754106	45.99	44.75	-7.85	49.56	29.09	69.72	-9.80	25.20	4.44	18.13	5.75	1110	0.671
60	UM0101058	01	SPECS	AK-968/13146122	35.23	63.07	11.13	67.69	32.12	85.23	-20.95	61.21	-6.53	54.44	5.92	1170	0.644
61	UM0096778	01	SPECS	AG-690/11634009	34.79	55.41	-3.90	28.94	28.84	66.72	-11.44	10.58	5.94	8.20	5.04	1218	0.607
62	UM0099861	01	SPECS	AK-778/40958947	57.06	92.90	34.60	89.55	25.31	91.53	-4.94	88.74	-31.50	86.36	8.40	1202	0.528
63	UM0093537	01	SPECS	AF-399/40772847	55.30	85.95	14.36	72.59	22.43	90.81	-31.39	74.12	-6.96	70.12	6.28	1310	0.534
64	UM0098333	01	SPECS	AG-690/40751449	54.01	89.49	23.13	74.11	26.54	78.16	-17.93	44.43	16.60	30.91	5.57	1198	0.316
65	UM0103856	01	SPECS	AM-814/41094541	61.32	62.29	18.29	63.96	36.24	55.25	66.45	5.21	9.99	52.39	1.27	757	0.017
66	UM0097001	01	SPECS	AG-690/11972457	50.52	77.46	22.17	64.38	37.47	87.78	-38.28	67.78	4.54	58.51	5.92	1233	0.617
67	UM0074324	02	SPECS	AK-968/37055100	31.29	37.41	6.83	16.36	22.95	61.79	1.09	8.90	10.69	2.61	4.67	1193	1.000
68	UM0007091	02	SPECS	AK-968/11165178	57.54	82.61	17.83	79.56	28.10	89.68	14.45	80.27	5.89	81.72	4.61	1032	0.381
69	UM0091821	01	SPECS	AA-768/34978021	77.38	86.93	18.32	72.42	49.85	91.91	-0.61	62.73	7.18	67.88	30.13	831	0.866
70	UM0100032	01	SPECS	AK-918/36937003	46.12	46.18	0.06	56.69	22.36	62.75	5.50	38.39	-28.95	58.72	5.83	1133	0.895
71	UM0007012	02	SPECS	AK-968/11164008	41.57	76.66	0.10	47.69	26.24	76.59	-2.74	22.97	-6.09	29.41	4.26	1195	0.620
72	UM0092622	01	SPECS	AE-848/32013001	59.39	89.58	12.98	64.73	40.47	93.63	-13.07	20.20	1.93	48.04	4.61	1133	0.374
73	UM0094671	01	SPECS	AG-205/33681021	63.62	64.67	17.70	53.74	61.07	86.87	14.84	43.80	12.53	60.89	4.74	1166	0.918
74	UM0012934	01	CHEMBRIDGE	5627239	57.14	79.85	-17.08	62.42	46.92	89.77	5.59	53.85	7.55	57.95	6.08	1066	0.726
75	UM0018211	01	CHEMBRIDGE	6104940	33.13	63.27	-20.12	46.85	24.23	69.66	0.50	24.45	-5.25	20.92	4.13	1028	0.913
76	UM0021111	01	CHEMBRIDGE	6444619	36.23	86.67	-25.66	71.84	28.13	79.68	2.98	24.94	1.10	21.27	4.63	1102	0.992
77	UM0028721	01	CHEMBRIDGE	7638598	34.94	70.82	11.65	66.48	23.10	68.69	0.66	31.04	-0.51	27.16	4.73	1061	1.000
78	UM0030219	01	CHEMBRIDGE	7661641	38.19	81.82	-6.39	47.50	20.02	52.04	5.19	-9.88	-1.19	-10.17	3.26	1158	0.714
79	UM0031037	01	CHEMBRIDGE	7677876	30.97	85.21	-33.90	85.58	33.17	75.37	14.03	16.72	0.90	17.29	4.67	1033	0.610
80	UM0033912	01	CHEMBRIDGE	7730387	58.62	83.13	-26.68	76.10	48.50	88.73	6.61	51.14	10.29	60.54	4.08	1079	0.293
81	UM0036576	01	CHEMBRIDGE	7783341	39.52	85.34	-49.88	66.67	35.26	82.85	1.40	43.11	2.04	36.38	4.88	943	0.540
82	UM0037150	01	CHEMBRIDGE	7797334	32.75	91.18	-7.30	87.22	20.83	87.73	-21.33	68.55	-4.05	66.89	5.46	1099	0.717
83	UM0035177	01	CHEMBRIDGE	7748948	65.55	95.99	31.78	73.09	67.35	91.06	2.84	47.17	8.72	47.08	5.53	1249	1.000
84	UM0006319	01	CHEMBRIDGE	5332028	49.16	88.80	42.57	87.02	25.03	87.25	-3.78	80.90	-15.37	79.45	6.12	906	0.901
85	UM0006445	01	CHEMBRIDGE	5335954	35.60	76.35	-55.12	85.87	23.46	82.55	0.97	72.99	-1.99	73.05	5.48	1057	0.997
86	UM0007481	01	CHEMBRIDGE	5356835	48.16	47.44	-19.74	57.89	22.94	62.97	-0.99	25.56	-5.93	22.95	5.12	1099	0.886
87	UM0021300	01	CHEMBRIDGE	6464887	61.67	64.02	-29.49	82.11	25.43	71.46	-2.68	11.01	-5.72	28.44	4.16	1175	0.798
88	UM0032114	01	CHEMBRIDGE	7693232	34.75	90.60	-36.49	84.35	24.24	91.70	-6.13	77.82	-9.37	82.92	4.57	1120	0.539
89	UM0024677	02	MAYBRIDGE	BTB 04957	65.89	83.32	-2.21	60.82	37.03	90.16	2.91	44.38	2.11	51.19	5.10	1080	0.576

					Primary screen Plk1-Map (stable)		Primary screen CRAF-BRAF (transient)		Confirmation Plk1-Map (stable)		Confirmation Plk1-Map (transient)		Confirmation CRAF-BRAF (transient)		Mitotic index		Co-IP
Cpd #	ID	Batch	Supplier	Supplier ID	% Inhibition	% Quench RLuc	% Inhibition	% Quench RLuc	% Inhibition	% Quench RLuc	% Inhibition	% Quench RLuc	% Inhibition	% Quench RLuc	Mitotic index (%)	Nb of analyzed cells	Values relative to top in plate column = 1
90	UM0026675	01	CHEMBRIDGE	7573944	31.54	93.69	-34.02	86.30	24.87	84.92	1.54	72.23	16.47	76.14	4.73	994	0.865
91	UM0040880	01	MAYBRIDGE	BTB 02165	31.42	80.31	-62.27	88.13	21.59	87.71	-6.44	81.17	-1.92	82.34	5.46	938	0.874
92	UM0024677	01	CHEMBRIDGE	7182303	31.73	89.49	-2.91	71.76	37.27	88.36	-7.35	35.36	-4.79	40.97	5.01	1114	1.000
93	UM0043471	01	MAYBRIDGE	CD 09636	45.16	88.06	-19.74	80.17	26.62	85.18	-8.24	58.20	-2.23	66.84	5.09	1026	0.841
94	UM0044029	01	MAYBRIDGE	DP 01660	51.69	94.69	3.78	88.15	44.21	87.91	35.12	87.25	17.09	86.44	4.83	1089	0.860
95	UM0045661	01	MAYBRIDGE	HTS 05195	45.78	92.59	22.52	68.30	24.98	86.69	-10.29	55.66	18.00	64.03	5.31	1028	0.688
96	UM0046655	01	MAYBRIDGE	HTS 10845	59.02	55.37	10.66	4.08	20.78	62.69	3.53	-18.74	11.95	-26.33	4.80	1011	0.422
97	UM0046929	01	MAYBRIDGE	HTS 12603	46.44	-15.66	-4.09	7.76	27.48	64.10	6.60	-1.90	10.94	-8.01	5.52	1064	0.924
98	UM0048554	01	MAYBRIDGE	KM 04938	61.42	73.36	9.17	35.83	30.73	72.21	11.50	-7.56	7.06	6.67	5.60	912	1.000
99	UM0053488	01	MAYBRIDGE	SCR 00880	64.82	96.93	-6.51	86.09	43.59	95.67	-1.55	69.68	-0.16	73.58	4.14	1035	0.535
100	UM0055888	01	MAYBRIDGE	SPB 06986	56.28	70.67	-5.98	49.29	21.68	81.71	36.75	83.83	10.77	85.00	3.46	1091	0.906
101	UM0057305	01	CHEMBRIDGE	7973027	58.20	71.17	11.87	24.48	25.59	81.25	-2.26	5.22	-1.25	8.14	4.90	1082	0.693
102	UM0057777	01	CHEMBRIDGE	7994213	53.01	86.51	-16.54	76.76	22.75	89.27	1.44	29.97	0.02	26.31	5.04	1156	0.955
103	UM0057973	01	CHEMBRIDGE	7998959	37.22	87.50	-4.16	82.45	23.14	87.15	-4.15	54.58	0.96	63.97	5.09	1269	0.873
104	UM0057989	01	CHEMBRIDGE	7999397	39.98	76.07	-14.54	44.82	21.84	74.15	3.12	3.89	2.23	7.27	4.60	1136	1.000
105	UM0061111	01	CHEMBRIDGE	9032660	34.88	91.43	-17.93	73.39	23.41	91.83	-7.42	66.08	-3.58	70.37	4.26	1151	0.913
106	UM0061877	01	CHEMBRIDGE	9037275	33.90	88.43	2.59	15.25	36.57	82.59	-5.15	17.65	4.63	4.64	4.67	1038	0.760
107	UM0063060	01	CHEMBRIDGE	9043703	35.84	92.37	-35.90	89.70	23.41	94.03	-6.99	88.59	3.94	89.06	5.67	1132	0.605
108	UM0064980	01	CHEMBRIDGE	9053935	49.87	77.46	1.34	75.29	27.61	91.79	-7.08	69.97	2.78	65.83	4.53	1115	0.353
109	UM0065245	01	CHEMBRIDGE	9055626	30.40	48.25	0.23	45.69	21.21	77.52	-4.20	43.98	-1.50	40.13	4.24	1303	0.457
110	UM0065246	01	CHEMBRIDGE	9055637	60.41	86.89	20.65	86.28	31.50	92.21	-16.97	73.64	1.99	74.92	4.62	1078	0.759
111	UM0067000	01	CHEMBRIDGE	5134355	57.76	73.12	25.91	66.49	36.70	62.66	11.76	35.42	17.99	39.64	4.74	1312	0.133
112	UM0067184	01	CHEMBRIDGE	5144173	57.92	71.84	0.00	55.88	32.29	74.81	7.79	10.94	5.72	32.74	5.62	1358	0.960
113	UM0074300	01	CHEMBRIDGE	5676580	67.59	86.55	27.85	82.94	50.01	93.10	15.89	81.59	14.86	87.48	5.03	1266	1.000
114	UM0126848	01	UdeM	UDM-000144	31.05	72.96	22.02	52.38	20.34	72.77	-4.60	17.86	-1.60	29.77	5.74	1089	0.824
115	UM0127131	01	UdeM	UDM-000770	62.96	77.03	35.58	89.67	22.78	80.34	-12.42	18.67	3.87	23.45	6.01	1152	0.644
116	UM0127134	01	UdeM	UDM-000775	73.20	82.73	20.93	83.07	45.22	85.88	-14.02	31.94	1.20	37.47	5.34	1046	0.427
117	UM0127135	01	UdeM	UDM-000776	65.86	88.45	29.57	86.80	42.50	88.81	-15.83	29.77	5.21	45.01	4.99	1161	0.526
118	UM0127153	01	UdeM	UDM-000933	45.02	65.77	-0.56	75.63	23.49	75.56	-12.10	25.09	-5.41	36.92	5.11	1229	0.926
119	UM0127154	01	UdeM	UDM-000934	69.73	78.13	30.28	81.67	28.42	83.23	-9.75	51.09	5.62	60.39	5.09	1122	0.917
120	UM0127191	01	UdeM	UDM-001223	47.30	72.72	25.58	69.47	25.29	76.88	-13.60	36.57	6.13	47.60	5.09	1163	1.000
121	UM0011080	01	CHEMBRIDGE	5541594	63.35	73.89	-6.16	36.21	38.10	75.39	-10.29	6.91	2.84	5.90	4.87	841	0.955
122	UM0052184	01	SPECS	AA-768/33245010	44.05	86.60	-8.30	44.16	30.57	79.14	-1.93	25.69	3.03	35.15	NT	NT	0.776

NT= Non tested

Table S3. Structure-Activity Relationship (SAR) analysis of Cpd 16. Chemical structure and IC_{50} values obtained in FP for the different analogs of Cpd 16 are presented.

Cpd #	UM number	Molecular mass	Molecular formula	Structure	FP - IC_{50} (µM)
**16**	UM0113574	407.79	$C_{16}H_{13}ClF_3NO_4S$	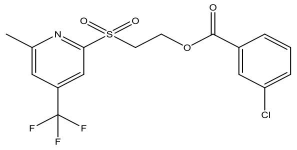	18.4 ± 10.2
**123**	UM0131803	373.35	$C_{16}H_{14}F_3NO_4S$	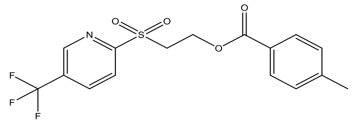	7.2
**124**	UM0131783	291.32	$C_{14}H_{13}NO_4S$	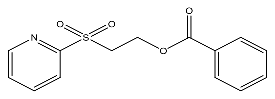	68.6 ± 18.5
**125**	UM0131784	393.76	$C_{15}H_{11}ClF_3NO_4S$	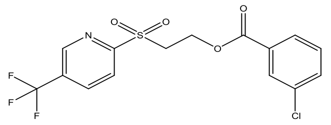	11.9 ± 7.2
**126**	UM0131785	360.21	$C_{14}H_{11}Cl_2NO_4S$	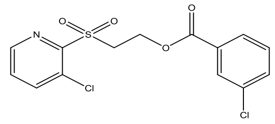	4.3
**127**	UM0131786	325.77	$C_{14}H_{12}ClNO_4S$	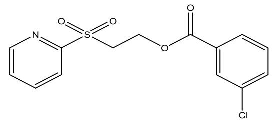	187.4
**128**	UM0131787	393.76	$C_{15}H_{11}ClF_3NO_4S$	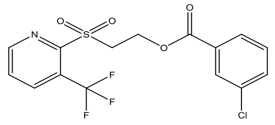	8.4
**129**	UM0041779	359.32	$C_{15}H_{12}F_3NO_4S$	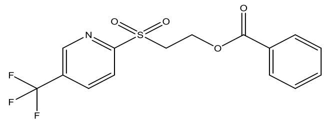	8.5
**130**	UM0113524	428.21	$C_{15}H_{10}Cl_2F_3NO_4S$	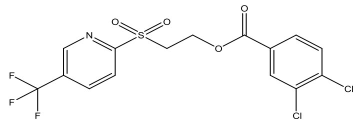	7.8
**131**	UM0113530	360.21	$C_{14}H_{11}Cl_2NO_4S$	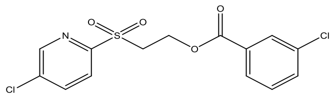	70.5
**132**	UM0113515	415.43	$C_{19}H_{20}F_3NO_4S$	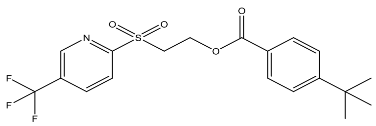	26.1
**133**	UM0113549	370.76	$C_{14}H_{11}ClN_2O_6S$	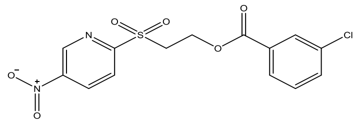	5.4
**15**	UM0113559	350.78	$C_{15}H_{11}ClN_2O_4S$	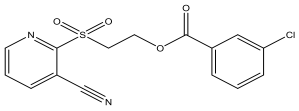	5.7 ± 2.8
**136**	UM0131792	293.34	$C_{14}H_{15}NO_4S$	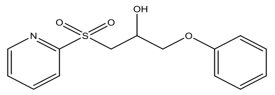	No inhibition
**137**	UM0131779	338	$C_{16}H_{16}ClNO_3S$	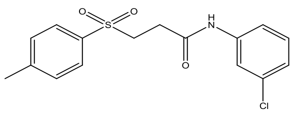	No inhibition

Cpd #	UM number	Molecular mass	Molecular formula	Structure	FP - IC$_{50}$ (μM)
**138**	UM0032816	387.28	$C_{17}H_{16}Cl_2O_4S$	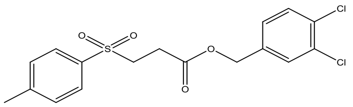	No inhibition
**139**	UM0131782	391.23	$C_{14}H_{12}Cl_2N_2O_5S$	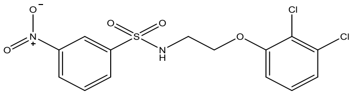	No inhibition
**140**	UM0131780	337.82	$C_{16}H_{16}ClNO_3S$	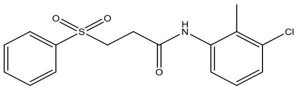	No inhibition
**141**	UM0131777	372.27	$C_{16}H_{15}Cl_2NO_3S$	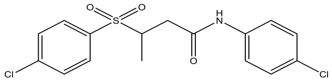	No inhibition
**142**	UM0041694	392.78	$C_{15}H_{12}ClF_3N_2O_3S$	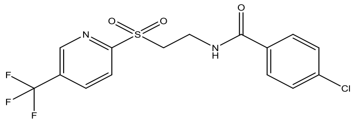	No inhibition
**143**	UM0131778	429.31	$C_{16}H_{10}F_7NO_3S$	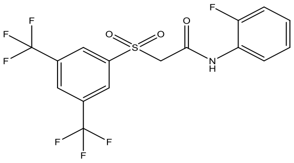	No inhibition
**144**	UM0113371	387.33	$C_{15}H_{12}F_3N_3O_4S$	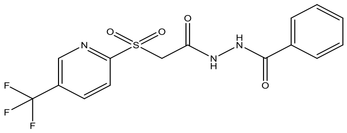	No inhibition
**145**	UM0109778	382.27	$C_{16}H_{16}BrNO_3S$	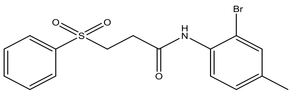	No inhibition
**146**	UM0131761	237.24	$C_9H_{10}F_3NOS$	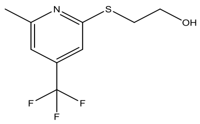	No inhibition
**147**	UM0131763	375.79	$C_{16}H_{13}ClF_3NO_2S$	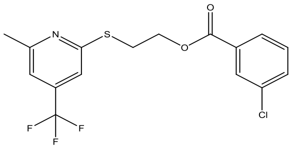	No inhibition
**148**	UM0131764	391.79	$C_{16}H_{13}ClF_3NO_3S$	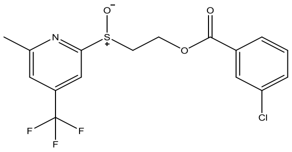	No inhibition
**149**	UM0131772	425.35	$C_{17}H_{13}F_6NO_3S$	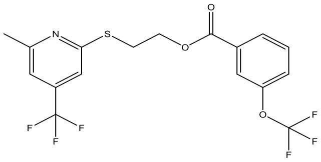	No inhibition
**150**	UM0131773	355.37	$C_{17}H_{16}F_3NO_2S$	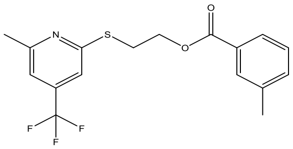	No inhibition
**151**	UM0131774	457.34	$C_{17}H_{13}F_6NO_5S$	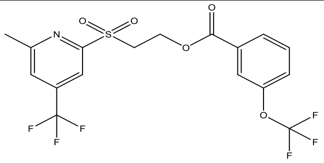	41.7

Cpd #	UM number	Molecular mass	Molecular formula	Structure	FP - IC_{50} (µM)
**152**	UM0131775	387.37	$C_{17}H_{16}F_3NO_4S$	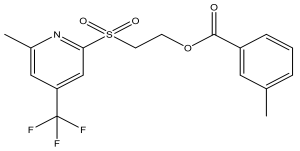	54.4
**153**	UM0131776	420.25	$C_{16}H_{13}BrF_3NO_2S$	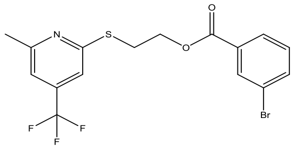	No inhibition
**154**	UM0131790	452.24	$C_{16}H_{13}BrF_3NO_4S$	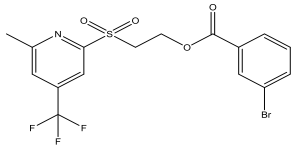	36.8
**155**	UM0131794	410.24	$C_{16}H_{12}Cl_2F_3NO_2S$	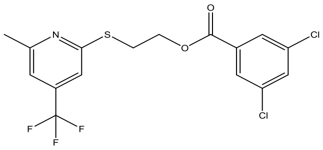	No inhibition
**156**	UM0131795	442.24	$C_{16}H_{12}Cl_2F_3NO_4S$	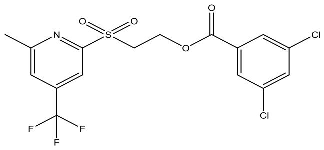	48.1
**157**	UM0131805	361.81	$C_{16}H_{15}ClF_3NOS$	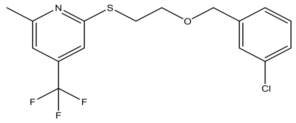	No inhibition
**158**	UM0131806	269.24	$C_9H_{10}F_3NO_3S$	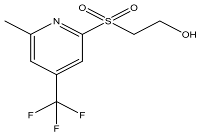	No inhibition
**159**	UM0131811	393.81	$C_{16}H_{15}ClF_3NO_3S$	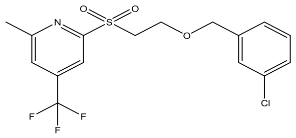	No inhibition
**160**	UM0131831	379.4	$C_{16}H_{20}F_3NO_4S$	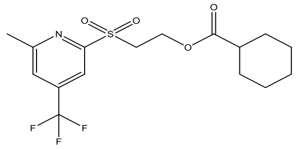	75.0
**161**	UM0131842	251.23	$C_9H_8F_3NO_2S$	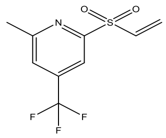	8.3 ± 2.6
**164**	UM0131845	347.33	$C_{10}H_{12}F_3NO_5S_2$	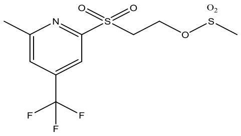	6.0
**168**	UM0131878	347.4	$C_{16}H_{20}F_3NO_2S$	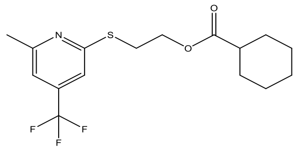	No inhibition
**169**	UM0132247	253.24	$C_9H_{10}F_3NO_2S$	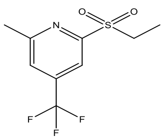	No inhibition
